# Protocol for quantifying chromatin DNA replication by multiplex labeling and single-molecule localization microscopy

**DOI:** 10.1016/j.xpro.2026.104882

**Published:** 2026-10-07

**Authors:** Zhiguang Xiao, Xiaodong Guan, Yujie Sun, Mengling Zhang, Qian Peter Su

**Affiliations:** 1 National Biomedical Imaging Center, College of Future Technology, Peking University, Beijing 100871, China; 2 State Key Laboratory of Membrane Biology & Biomedical Pioneering Innovation Center (BIOPIC), Peking University, Beijing 100871, China; 3 School of Life Sciences, Peking University, Beijing 100871, China; 4 Department of Molecular Biology and Genetics, Cornell University, Ithaca, NY 14853, USA; 5 School of Biomedical Engineering, University of Technology Sydney, Sydney, NSW 2007, Australia

**Keywords:** Biophysics, Cell Biology, Genomics

## Abstract

We present a protocol for imaging and quantifying chromatin DNA replication-associated signals in synchronized mammalian cells. We describe steps for cell-cycle enrichment, nucleoside analog-based metabolic labeling, and fluorescence detection. We then detail the procedure for dual-color STORM imaging and SR-Tesseler-based cluster analysis. It can be adapted to distinct questions in DNA replication biology by modifying synchronization schemes, pulse durations, labeling strategies, fluorescence detection channels, or analysis parameters.

For complete details on the use and execution of this protocol, please refer to Zhang et al.^1^

## Highlights

• Workflow for enriching mammalian cells at defined cell-cycle windows
• Steps for nucleoside analog-based labeling of nascent chromatin DNA synthesis
• Steps for fluorescence detection of chromatin DNA labels and proteins of interest
• Guidance on dual-color STORM imaging and spatial quantification of replication signals

## Abstract (editor highlights)

Publisher’s note: Undertaking any experimental protocol requires adherence to local institutional guidelines for laboratory safety and ethics.

## Graphical abstract

**Figure:**
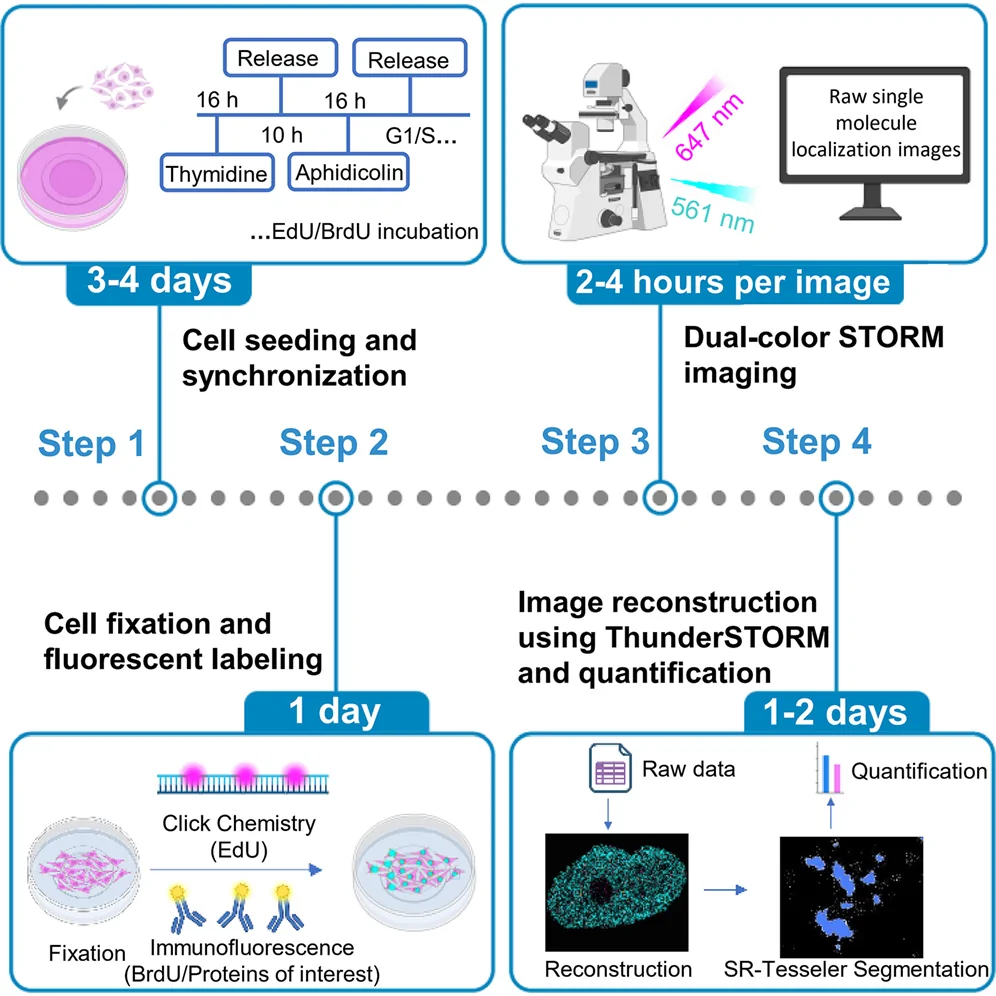

## Before you begin

Accurate control of DNA replication is essential for faithful genome duplication and maintenance of genome stability.^2^^,^^3^ Disruption of this process can cause replication stress, DNA damage, and chromosomal instability, and is therefore closely linked to cancer and other genome instability-associated diseases.^3^^,^^4^ Because replication is coordinated within higher-order chromatin and nuclear architecture, methods that can resolve its spatial organization and activity are important for understanding how replication is regulated in different cellular states.^5^^,^^6^^,^^7^ Conventional diffraction-limited fluorescence microscopy typically provides a lateral resolution of approximately 200–250 nm, which may be insufficient to distinguish closely spaced replication-associated foci as separate structures.^8^^,^^9^ By contrast, under appropriate labeling and acquisition conditions, single-molecule localization microscopy can localize individual fluorophore-emission events with a lateral precision of several tens of nanometers, enabling the resolution and quantitative analysis at the level of individual replication-associated foci, including the resolution of closely adjacent foci. Therefore, STORM imaging is needed to distinguish adjacent replication foci and quantify the nanoscale spatial organization of DNA replication.^1^

Many replication-related questions require cells to be enriched at defined cell-cycle windows before labeling and imaging.^1^^,^^10^^,^^11^ The synchronization strategy, release duration, and labeling window should be selected according to the biological question being addressed. For example, some experiments may require enrichment near the transition into DNA synthesis, whereas others may compare different periods of S phase or examine replication-associated signals after genetic, chemical, or environmental perturbations. These parameters should be optimized for each cell type and experimental condition before downstream super-resolution imaging.

The protocol below is organized as a practical workflow for labeling replication-associated DNA synthesis in defined cell-cycle windows and correlating these signals to fixed-cell fluorescence imaging and quantitative spatial analysis. It can be used to examine localization density, number of localization events, distribution, clustering, and spatial relationships, and can be combined with immunofluorescence labeling to relate DNA synthesis patterns to chromatin organization, nuclear architecture, replication-associated proteins, or DNA damage responses.

### Innovation

This protocol provides an integrated strategy to convert temporally controlled DNA synthesis labeling into nanoscale spatial information in fixed cells. A key advantage is that replication-associated signals captured in defined pulse windows can be directly compared with DNA or protein markers in the same imaging context. Coupling dual-color STORM with SR-Tesseler-based segmentation further enables standardized quantification of signal number, size, density, distribution, and the spatial relationships between replication-associated localization clusters detected in the two fluorescence channels. This design makes the workflow suitable for analyzing the nanoscale organization of replication-associated signals under different cellular states or perturbation conditions.

### Cell culture and seeding

**Timing: 1 day**

This section describes the culture and seeding of HeLa cells on glass-bottom dishes for subsequent synchronization, labeling, and STORM imaging.

***Note:*** This cell culture and seeding procedure is generally applicable to adherent mammalian cells, but the seeding density should be optimized according to the proliferation rate and adhesion properties. In the example workflow described here, HeLa cells are maintained in 10 cm dishes in complete DMEM supplemented with 10% fetal bovine serum and 100 U/mL penicillin-streptomycin at 37°C with 5% CO_2_. Cells are passaged every 2–3 days at approximately 80% confluence using 0.25% trypsin. For imaging experiments, cells are seeded on 35 mm glass-bottom dishes with No. 1.5 coverglass (approximately 0.17 mm thick) at a density that yields 20%–30% confluence on the next day. Optional poly-L-lysine coating can improve cell adhesion during repeated washing and staining steps.

1. Coat each sterile 35 mm glass-bottom dish with 0.1% poly-L-lysine (PLL).
  a. Add enough PLL solution to completely cover the glass area, typically 300–500 μL per dish.
  b. Incubate at 20°C–25°C for 20 min.
2. Aspirate the PLL solution completely and rinse the dish twice with cell-culture-grade distilled water to remove excess coating solution.
3. Allow the dish to air-dry in a biosafety cabinet for 15–30 min before cell seeding.
4. Warm complete DMEM, PBS, and 0.25% trypsin to 37°C before use.
5. Aspirate the old medium from an exponentially growing 10 cm culture dish of HeLa cells and wash the cells twice with PBS, using enough volume to fully cover the dish surface.
6. Add 1 mL of 0.25% trypsin and incubate at 37°C for 2 min, or until the cells detach. Monitor cell detachment under a microscope.
7. Neutralize trypsin by adding 4–5 mL of complete DMEM and gently pipette up and down to obtain a uniform single-cell suspension.
8. Transfer the cell suspension to a 15 mL tube and centrifuge at 300 × *g* for 2 min.
  a. Remove the supernatant, resuspend the pellet in complete DMEM.
  b. Wash the cells twice more in the same way to remove residual trypsin.
9. Seed cells onto the PLL-coated 35 mm glass-bottom dish in 2 mL of complete DMEM at a density that will yield 20%–30% confluence on the next day.

***Note:*** The exact seeding density should be empirically optimized according to the growth rate of the cell line and experimental conditions.

10. Gently swirl the dish to distribute cells evenly across the glass surface and incubate for 12–16 h at 37°C in a humidified atmosphere containing 5% CO_2_.

**CRITICAL:** Do not allow the cells to become overly confluent before synchronization. High cell density can affect synchronization efficiency, compromise image quality, and complicate single-cell segmentation during downstream analysis.

**CRITICAL:** This protocol has been experimentally validated in HeLa cells. Application to primary cells, stem cells, or other cell types requires independent optimization of seeding density, synchronization strategy, drug concentration and exposure time, release timing, pulse duration, and labeling and detection conditions.

### Preparation of dye-conjugated secondary antibody

**Timing: 2–3 h**

This section describes the in-house preparation and purification of a Cy3B-conjugated secondary antibody for immunofluorescence staining and STORM imaging. The conjugation procedure and representative purification outcomes are shown in Figure 1.

***Note:*** Although commercially available dye-conjugated secondary antibodies can be used after validation, in this example, we prepared the Cy3B-conjugated secondary antibody in-house to improve quantitative consistency. Using the same antibody stock and dye across experiments helps reduce batch-to-batch variation. After purification, the labeling quality can also be checked by absorbance measurement. This is useful for STORM-based quantification because differences in labeling efficiency or dye performance may affect fluorescence intensity, localization density, and segmented signal clusters.

**Figure 1:**
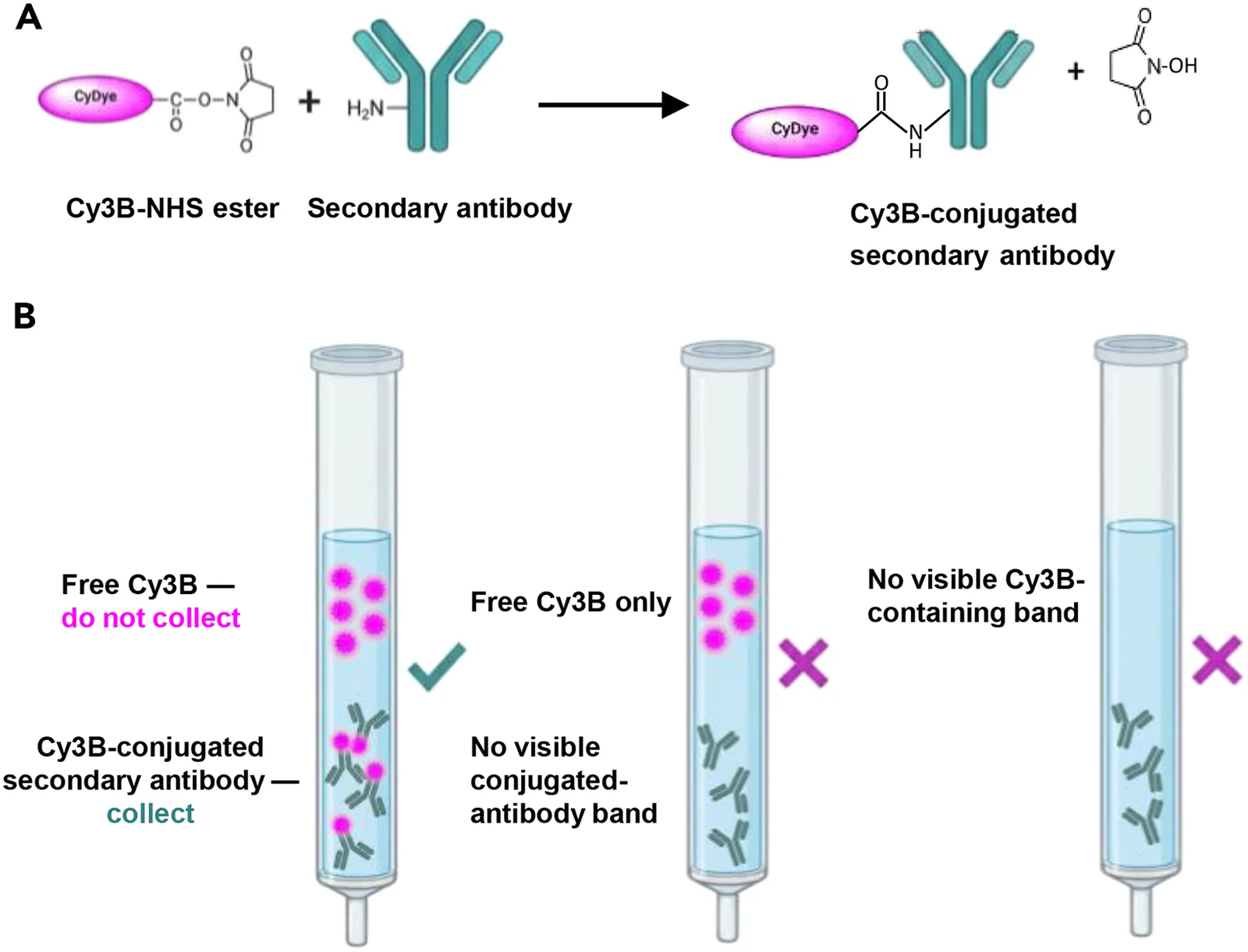
Preparation and purification of Cy3B-conjugated secondary antibody (A) Schematic illustration of NHS ester-mediated labeling of the secondary antibody. The NHS ester group of Cy3B reacts with primary amine (NH_2_) groups to form a stable covalent linkage. In this workflow, Cy3B-NHS ester was reacted with a secondary antibody to generate Cy3B-conjugated secondary antibody. (B) Schematic of the expected band patterns during gel-filtration purification. In the successful case, two Cy3B-containing bands are visible. The lower leading band corresponds to Cy3B-conjugated secondary antibody and should be collected. The upper trailing band corresponds to free Cy3B and should not be collected. The presence of only the free-Cy3B band indicates that no visible conjugated-antibody band was obtained. The absence of any visible Cy3B-containing band indicates a possible problem with sample loading or recovery.

Prepare the Cy3B-labeling reaction mixture according to the recipe in materials and equipment.

11. Mix the labeling reaction gently and incubate for 30 min at 20°C–25°C, protected from light, on a shaker.
12. During the reaction, equilibrate one NAP-5 gel filtration column for each labeling reaction by passing three column volumes (750 μL) of PBS through the column.

***Note:*** NAP-5 gel filtration is used to separate the high molecular weight Cy3B-conjugated secondary antibody from unreacted low molecular weight Cy3B-NHS dye and to exchange the reaction buffer for PBS before downstream use.

13. Add the entire reaction mixture to the center of the column.
14. Allow the sample to fully enter the resin bed. After the last drip appears, add 550 μL PBS to wash the column.
15. Add 300 μL PBS and collect the eluate into a 1.5 mL microcentrifuge tube.
16. Measure the purified Cy3B-conjugated secondary antibody using the Proteins & Labels application on a NanoDrop spectrophotometer to determine the antibody concentration and the molar dye-to-antibody ratio.

***Note:*** Cy3B-NHS ester labels primary amines on the secondary antibody to form stable covalent conjugates. A dye-to-antibody ratio of approximately 2–4 is typically suitable for fluorescence imaging. The molar dye-to-antibody ratio represents the average number of Cy3B molecules conjugated per antibody molecule.

17. Aliquot the purified Cy3B-conjugated secondary antibody, add sterile glycerol to a final concentration of 40%, and store at −20°C protected from light.

***Note:*** Use one NAP-5 column for each labeling reaction. The expected eluate should contain dye-conjugated antibody rather than free dye alone or unlabeled protein alone.

Representative purification outcomes are schematically shown in Figure 1B. In a successful separation, the lower leading band contains Cy3B-conjugated secondary antibody and should be collected, whereas the upper trailing band contains free Cy3B and should not be collected. A single visible band suggests that the protein was present but conjugation was unsuccessful, whereas the absence of a visible band suggests that the protein was not successfully recovered from the column. A previously validated commercial labeled secondary antibody can be used as a positive control when establishing this workflow. This workflow can also be adapted for labeling other purified proteins after optimization.

## Key resources table

**Table:** 

REAGENT or RESOURCE	SOURCE	IDENTIFIER
**Antibodies**		

Mouse anti-BrdU (B44)	BD Biosciences	Cat# 347580; IF dilution: 1:50
Rabbit anti-PCNA	Cell Signaling Technology	Cat# 13110; IF dilution: 1:200
Donkey anti-mouse IgG	Jackson ImmunoResearch	Cat# 715-005-151; IF dilution after Cy3B conjugation: 1:50

**Chemicals, peptides, and recombinant proteins**		

Dulbecco’s Modified Eagle Medium (DMEM)	Gibco	Cat# 11965-092
Fetal Bovine Serum (FBS)	Gibco	Cat# 10099-141
Penicillin-Streptomycin	Gibco	Cat# 15140-122
Trypsin (0.25%)	Gibco	Cat# 25200-056
PBS	Gibco	Cat# C10010500BT
Poly-L-Lysine	Sigma-Aldrich	Cat# P8920-100ML
Thymidine	Sigma-Aldrich	Cat# T1895
Aphidicolin	Sigma-Aldrich	Cat# A0781
BrdU	Sigma-Aldrich	Cat# B5002
Bovine Serum Albumin (BSA)	Sigma-Aldrich	Cat# A7906
MgCl_2_	Sigma-Aldrich	Cat# M2393
NaHCO_3_	Sigma-Aldrich	Cat# S5761
Glycerol	Sigma-Aldrich	Cat# G5516
Glucose	Sigma-Aldrich	Cat# G7021
NaCl	Sigma-Aldrich	Cat# S5886
Tris-HCl	Sigma-Aldrich	Cat# T5941
HCl	Sigma-Aldrich	Cat# H1758
PIPES	Sigma-Aldrich	Cat# P6757
EGTA	Sigma-Aldrich	Cat# E3889
DMSO	Sigma-Aldrich	Cat# D8418
NaN_3_	Sigma-Aldrich	Cat# S2002
β-mercaptoethanol	Sigma-Aldrich	Cat# M6250
Glucose oxidase	Sigma-Aldrich	Cat# G2133
Catalase	Sigma-Aldrich	Cat# C9322
Cy3B-NHS dye	Cytiva	Cat# PA63101
NAP-5 column	Cytiva	Cat# 17-0853-01
Triton X-100	Sigma-Aldrich	Cat# T8787
Formaldehyde	Sigma-Aldrich	Cat# F8775

**Critical commercial assays**		

Click-iT EdU Imaging Kit	Thermo Fisher Scientific	Cat# C10340

**Experimental models: Cell lines**		

HeLa S3 cells	Kind gift from Dr. Wei Guo, University of Pennsylvania	N/A

**Software and algorithms**		

Fiji	Schindelin et al., 2012^12^	version 2.14.0/1.54f; https://fiji.sc/
ThunderSTORM	Ovesný et al., 2014^13^	version 1.3; https://zitmen.github.io/thunderstorm/
SR-Tesseler	Levet et al., 2015^14^	version 1.0; https://github.com/flevet/SR-Tesseler/releases

**Other**		

35 mm glass-bottom dish	Cellvis	Cat# D35-20-1.5-N
Adhesive sample coverslip film for adherent samples	Sunbloss	HXSP008
TetraSpeck Microspheres	Thermo Fisher Scientific	Cat# T7279
Olympus IX83 microscope	Olympus	IX83
100× oil-immersion objective lens	Olympus	UPlanXApo100XO NA 1.45
Multi-band dichroic mirror	Chroma	ZT405/488/561/647rpc
Multi-band emission filter	Chroma	ZET405/488/561/647m
561 channel emission filter	Semrock	FF01-585/40-25
647 channel emission filter	Chroma	ET690/70m
EMCCD camera	Andor	iXon+ DU897
405 nm laser	Coherent	OBIS
561 nm laser	MPB Communications	2RU-VFL-P-1000-561-B1R
647 nm laser	MPB Communications	2RU-VFL-P-1000-647-B1R
Near-infrared focus-lock laser	Thorlabs	LP940-SF30

## Materials and equipment

### Preparation of dye-conjugated secondary antibody

**Table:** Preparation of secondary antibody stock

Reagent	Stock concentration	Storage	Final concentration	Volume
Secondary antibody	1.0–1.3 mg/mL	4°C	0.5–0.6 mg/mL	1 mL
Glycerol	80%	−20°C	40%	1 mL
NaN_3_	10%	−20°C	0.05%	10 μL

***Note:*** Aliquot the stock solution into 100 μL or 150 μL portions and store at −20°C for up to 12 months. Select the appropriate secondary antibody based on the host species of the primary antibody used for staining. Commercially available donkey anti-mouse, anti-rabbit, or anti-rat secondary antibodies can be used as starting materials.

***Note:*** Sodium azide is included as a preservative to limit microbial growth during storage of aqueous antibody stocks. Sodium azide is not required when the antibody preparation is used immediately.

**Table:** Cy3B labeling reaction recipe

Reagent	Stock concentration	Storage	Final concentration	Volume
Secondary antibody stock	∼0.5 mg/mL, ∼3.3 μM	−20°C	0.25 mg/mL, ∼1.7 μM	∼100 μL
NaHCO_3_	1 M, freshly prepared	−20°C	0.1 M	20 μL
Cy3B-NHS dye	500–1000 μM	−20°C	50–100 μM	20 μL
PBS	1×	20°C–25°C	1×	up to 200 μL

***Note:*** Combine the secondary antibody, Cy3B-NHS dye, and NaHCO_3_, and adjust the final volume to 200 μL before gel filtration. Protect the reaction from light throughout the labeling procedure. Mildly alkaline conditions are important for efficient NHS ester coupling. A pH around 8.0 is recommended. As illustrated in Figure 1A, the NHS ester group of Cy3B reacts with primary amines on the secondary antibody to form a stable covalent conjugate. In principle, NHS ester chemistry can also be applied to other amine-containing biomolecules, including peptides, proteins, antibodies, nucleic acids, and small molecules. After purification, aliquot the Cy3B-conjugated secondary antibody and store at −20°C, protected from light, for up to 12 months.

### Reagents and solutions for synchronization and nucleoside analog labeling

**Table:** Reagents for cell-cycle synchronization and DNA labeling

Reagent	Stock concentration	Working concentration	Solvent
Thymidine	200 mM (100×)	2 mM	sterile water
Aphidicolin	200 μg/mL (100×)	2 μg/mL	DMSO
EdU	10 mM (1000×)	10 μM	DMSO
BrdU	16 mM (1000×)	16 μM	DMSO

Prepare thymidine, aphidicolin, EdU, and BrdU stock solutions in advance, aliquot them to avoid repeated freeze-thaw cycles, and prepare fresh working solutions in pre-warmed complete DMEM on the day of use. Thymidine and EdU stock solutions can be stored for up to 12 months. Aphidicolin stock solution can be stored for up to 6 weeks. BrdU stock solution can be stored for up to 6 months.

**Table:** Extraction buffer for pre-fixation treatment

Reagent	Stock concentration	Final concentration	Amount for 50 mL
PIPES, pH 7.0	1 M	0.1 M	5 mL
EGTA	0.1 M	1 mM	0.5 mL
MgCl_2_	1 M	1 mM	50 μL
Triton X-100	10%	0.2%	1 mL
ddH_2_O	N/A	N/A	to 50 mL

• Prepare the extraction buffer base without Triton X-100 in advance and store at 4°C for up to 1 month.
• Immediately before use, add 20 μL of 10% Triton X-100 to 1 mL of extraction buffer base to obtain a final concentration of 0.2%, and mix gently to avoid bubbles.
• Use the freshly prepared extraction buffer on the same day.

### STORM imaging buffer

STORM imaging is performed in a freshly prepared imaging buffer consisting of standard imaging buffer (STIB), glucose oxidase-catalase stock solution (GLOX), and β-mercaptoethanol (β-ME). In this workflow, β-ME was selected because it supported suitable fluorophore blinking under the described imaging conditions. Mercaptoethylamine (MEA) is a commonly used alternative reducing agent. The choice and concentration of the reducing agent should be optimized for the fluorophore, imaging buffer, and microscope system.

**Table:** Standard imaging buffer (STIB)

Reagent	Final concentration	Amount for 500 mL
Glucose	10% (w/v)	250 mL of 20% glucose solution
NaCl	20 mM	0.585 g or 2 mL of 5 M NaCl
Tris-HCl, pH 8.0	100 mM	50 mL of 1 M stock
Ultrapure water	N/A	to 500 mL

• Prepare STIB in ultrapure water and adjust the pH to 8.0 with HCl or NaOH.
• Filter the solution through a 0.22 μm filter to remove bacteria and precipitates.
• Degas the filtered solution under vacuum for 12–16 h.
• Aliquot the degassed buffer into airtight 2 mL tubes, taking care to minimize air introduction during aliquoting.
• Store at 4°C. Properly prepared STIB can be stored for 3 months, but discard the solution if turbidity or precipitates appear before use.

**Table:** GLOX stock solution

Reagent	Final concentration	Amount for 2 mL
Glucose oxidase	60 mg/mL	0.12 g
Catalase	6 mg/mL	0.012 g
80% glycerol	40% (v/v)	1 mL
PBS	N/A	1 mL

• Prepare 80% glycerol by mixing 32 mL glycerol with 8 mL ddH_2_O. Mix gently and allow bubbles to dissipate naturally.
• Dissolve glucose oxidase and catalase first in 1 mL PBS, then add 1 mL of 80% glycerol.
• Centrifuge the solution for 2 min at 20,000 × *g* and collect only the yellow supernatant for use.
• Aliquot the clarified supernatant into 200 μL portions in 1.5 mL tubes.
• Store aliquots at −20°C for up to 3 months. After thawing, keep the working aliquot at 4°C and monitor its appearance before use. Discard the solution if it becomes turbid or if visible precipitates remain after centrifugation.
• In this workflow, GLOX is used as an oxygen-scavenging system to reduce dissolved oxygen and support fluorophore switching during single-molecule localization microscopy. Its requirement and concentration should be optimized when alternative fluorophores, reducing agents, or imaging-buffer formulations are used.

**Table:** Working STORM imaging buffer

Reagent	Final concentration	Amount for 2.04 mL working buffer
STIB	N/A	2 mL
GLOX stock solution	∼600 μg/mL glucose oxidase, ∼60 μg/mL catalase	20 μL
β-ME	1% (v/v)	20 μL

• Bring STIB to 20°C–25°C before preparing the working imaging buffer.
• Prepare the working imaging buffer immediately before use by adding 20 μL GLOX stock solution and 20 μL β-ME to 2 mL STIB.
• Mix gently and avoid introducing bubbles.
• Use the working buffer within approximately 2 h after preparation.

**CRITICAL:** β-ME is toxic and volatile. Prepare the working imaging buffer in a fume hood and wear appropriate personal protective equipment. Minimize air exposure and avoid vigorous mixing, as oxygen introduction and bubble formation can reduce imaging performance.

## Step-by-step method details

### Replication-associated metabolic labeling in synchronized cells

**Timing: 2–3 days**

This section describes synchronization of HeLa cells, release into a defined replication window, and pulse labeling with nucleoside analogs for downstream fluorescence imaging.

***Note:*** The goal of this section is to describe how cells can be enriched at a selected cell-cycle stage, released into a defined replication window, and pulse-labeled with nucleoside analogs for downstream fluorescence imaging and spatial analysis. This strategy can be adapted to distinct questions in DNA replication biology by changing the synchronization method, release duration, pulse length, or nucleoside analog combination.

Replication studies often require cells to be compared at specific cell-cycle or S-phase windows. For example, cells can be enriched near the G1/S transition and then released into S phase, or release timing can be adjusted to examine different S-phase stages. In the example workflow below, HeLa cells are synchronized at G1/S transition using a thymidine block, release, and aphidicolin block as outlined in Figure 2A. Sequential thymidine and aphidicolin treatment was used to improve synchronization efficiency in HeLa cells before nucleoside-analog labeling.^11^^,^^15^ This design is used as a representative workflow and should be re-optimized when applied to other cell lines, perturbations, or labeling windows.

1. Inspect the cells seeded on PLL-coated 35 mm glass-bottom dishes and confirm that they are healthy, evenly distributed, and at approximately 20%–30% confluence.
2. Prepare the thymidine-containing medium by adding 20 μL of 200 mM thymidine stock to 1.98 mL complete DMEM.
  a. Mix thoroughly by gentle pipetting or swirling before use to ensure even distribution of the drug.
  b. Aspirate the culture medium and add 2 mL of pre-warmed complete DMEM containing 2 mM thymidine to each dish.
3. Incubate the cells for 16 h at 37°C in a humidified incubator with 5% CO_2_.
4. Remove the thymidine-containing medium and wash the cells three times with pre-warmed PBS.
  a. During each wash, add enough PBS to fully cover the glass surface.
  b. Gently swirl the dish thoroughly after each PBS addition to ensure efficient removal of residual thymidine and complete release from the block.
  c. Aspirate the PBS carefully after each wash to avoid detaching cells from the glass surface.
  d. Completely remove residual PBS after the final wash.
5. Add fresh pre-warmed complete DMEM and incubate the cells for 10 h at 37°C with 5% CO_2_.
6. Replace the medium with 2 mL of pre-warmed complete DMEM containing 2 μg/mL aphidicolin (20 μL of 200 μg/mL stock in 1.98 mL medium).
7. Incubate the cells for 16 h at 37°C in a humidified incubator with 5% CO_2_.
8. Examine the cells under a microscope before release. Proceed only when the cells remain attached and show no obvious signs of excessive rounding or detachment.
9. Remove the aphidicolin-containing medium and wash the cells three times with pre-warmed PBS as described in step 4, taking care to keep the washing procedure and timing consistent across all samples.
10. Release the cells into the selected labeling window by adding 2 mL of pre-warmed complete DMEM containing the selected nucleoside analog.
11. Incubate the cells at 37°C in a humidified incubator with 5% CO_2_ for the selected pulse-labeling duration.
12. Remove the nucleoside analog-containing medium and proceed according to the experimental design.
  a. For experiments with a single labeling window, quickly wash the cells once with pre-warmed PBS to remove residual labeling reagent, and immediately proceed to step 13.
  b. For experiments requiring an additional labeling window, wash the cells three times with pre-warmed PBS to remove residual labeling reagent.
    i. After the final wash, repeat steps 5–11 using another nucleoside analog.
    ii. After the second pulse, quickly wash the cells once with pre-warmed PBS and immediately proceed to step 13.

***Note:*** Different nucleoside analogs can be selected according to the experimental design and detection method. EdU is commonly detected by click chemistry, whereas BrdU, IdU, and CldU are usually detected by antibody-based staining after DNA denaturation. When multiple analogs are used in the same workflow, antibody specificity, spectral compatibility, pulse duration, and staining order should be validated before formal imaging experiments.

***Note:*** The 45-min BrdU and 10-min EdU labeling windows were selected based on our previous study.^11^ The 45-min BrdU pulse was used to label early replication domains while retaining distinguishable domain boundaries, whereas the 10-min EdU pulse was used to label replication initiation sites. These interpretations depend on synchronization efficiency, pulse duration, detection strategy, and downstream image analysis, and should be re-evaluated when the workflow is adapted to other questions.

***Note:*** Synchronization efficiency should be validated, for example by flow cytometry using DNA content analysis with propidium iodide (PI) or DAPI staining. A detailed procedure for cell-cycle analysis by flow cytometry is provided in our previous study.^11^ This validation helps confirm that the majority of cells are enriched in the intended cell-cycle window before interpreting BrdU- or EdU-labeled replication patterns.

13. Remove the culture medium and gently wash the cells once with PBS.
  a. Immediately add 1 mL of freshly prepared extraction buffer (see materials and equipment) to each dish.
  b. Incubate for 30 s at 20°C–25°C.

***Note:*** This brief pre-extraction step removes a portion of the soluble nucleoplasmic background before fixation and helps improve contrast of replication-associated structures during downstream super-resolution imaging.

**CRITICAL:** Add Triton X-100 to the extraction buffer immediately before use. Do not over extract the cells, as prolonged extraction can disrupt nuclear morphology and reduce retention of replication-associated structures. The 30 s extraction time was suitable for HeLa cells grown on glass-bottom dishes under the conditions used here and should be re-optimized if cell density, substrate coating, or cell type is changed.

14. Immediately remove the extraction buffer and wash the cells once with PBS before fixing the cells with 1 mL of 4% formaldehyde for 10 min at 20°C–25°C.
15. Remove the fixation solution and wash the cells three times with PBS before proceeding to click chemistry and immunofluorescence.

**CRITICAL:** Maintain identical cell density, synchronization duration, wash timing, and release timing across all samples.

**Figure 2:**
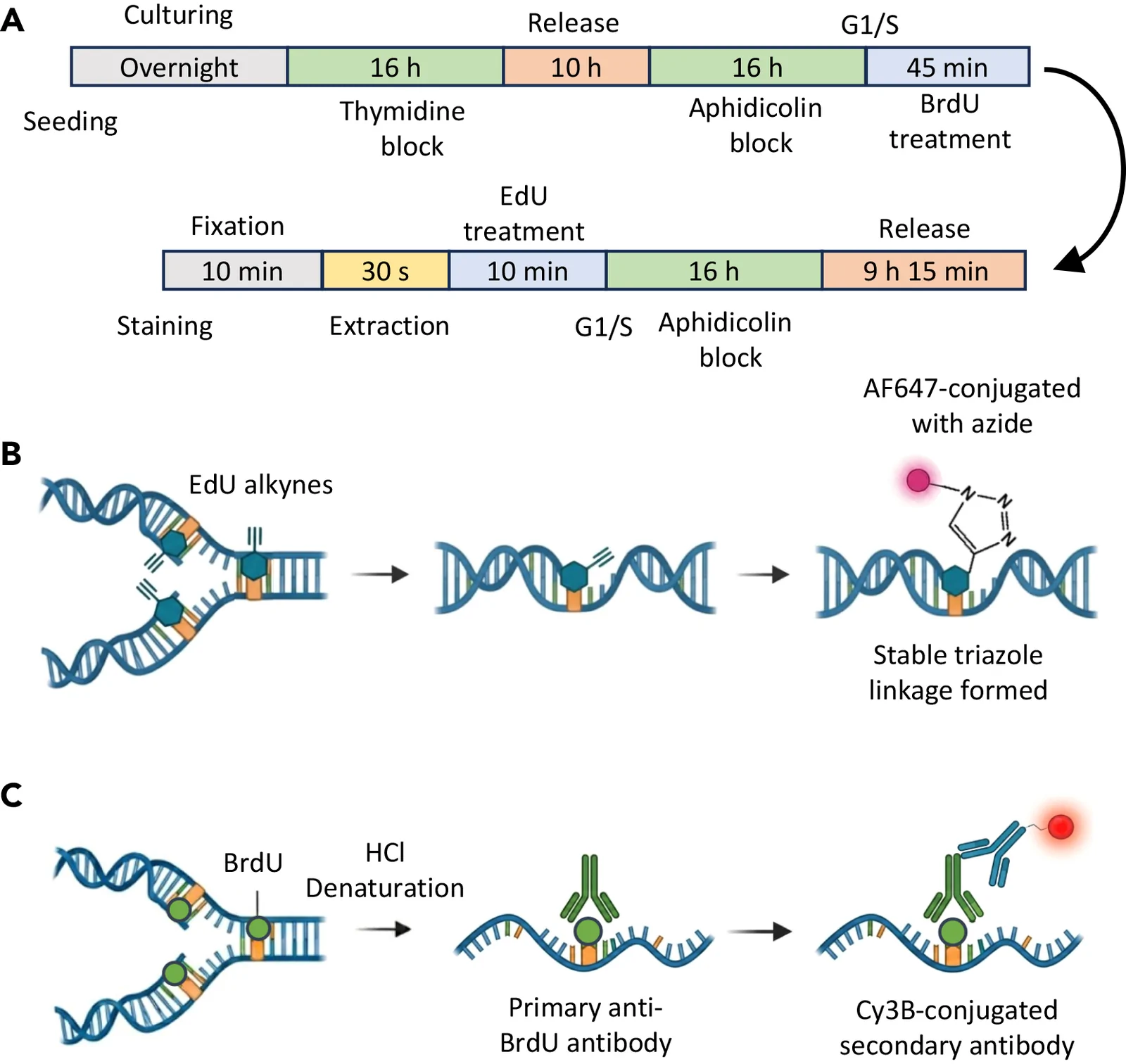
Schematic overview of synchronization, EdU click labeling, and BrdU immunofluorescence detection (A) Workflow for sequential cell synchronization. Cells were seeded and cultured for 12–16 h, followed by a 16 h thymidine block, 10 h release, and 16 h aphidicolin block. After release into early S phase, cells were labeled with BrdU for 45 min. Cells were then released for 9 h 15 min, subjected to a second 16 h aphidicolin block, and pulse-labeled with EdU for 10 min after release into early S phase. Samples were subsequently processed for extraction, fixation, and downstream fluorescence labeling. (B) Principle of EdU click labeling. EdU incorporated into newly synthesized DNA contains an alkyne group, which reacts with an azide-conjugated fluorophore through copper-catalyzed azide–alkyne cycloaddition, resulting in the formation of a stable triazole linkage. (C) Principle of BrdU immunofluorescence detection. BrdU incorporated into replicating DNA is recognized by a primary anti-BrdU antibody, followed by detection with a Cy3B-conjugated secondary antibody to generate a fluorescent signal.

### Fluorescence labeling of fixed samples for replication imaging

**Timing: 1–2 days, including 12–16 h primary antibody incubation at 4°C**

This section describes click chemistry and antibody-based detection of metabolically incorporated nucleoside analogs in fixed cells.

***Note:*** In this protocol, fixed-cell labeling includes two related modules: detection of metabolically incorporated nucleoside analogs and immunofluorescence detection of proteins. The first module reports DNA synthesis during the selected labeling windows, whereas the second module can be used to examine replication-associated proteins, chromatin regulators, nuclear structural proteins, or DNA damage markers.

In the workflow described here, click chemistry is performed before acid denaturation and antibody-based nucleoside analog detection. This order preserves click-labeling efficiency while allowing subsequent exposure of halogenated nucleoside analog epitopes for antibody recognition. If additional protein markers are included, staining conditions should be optimized in pilot experiments because acid denaturation can affect some protein epitopes.

16. Add 300 μL blocking buffer consisting of PBS containing 5% BSA and 0.5% Triton X-100 to each 35 mm glass-bottom dish and incubate for 30 min at 20°C–25°C. This volume is sufficient to cover the glass-bottom imaging area.

***Note:*** For convenience, prepare 10% BSA in PBS in advance, aliquot into 500 μL portions, and store at −20°C for up to 3 months. Before use, thaw one aliquot and mix with 450 μL PBS and 50 μL 10% Triton X-100 to obtain 1 mL blocking buffer containing 5% BSA and 0.5% Triton X-100.

17. Wash the samples once with PBS for 5 min on a shaker.
18. Prepare the click chemistry reagents according to the manufacturer’s instructions.
  a. Reconstitute the fluorescent azide reagent with the recommended solvent.
  b. Prepare the reaction buffer and buffer additive as instructed.
  c. Store the remaining stock solutions according to the manufacturer’s recommendations.
19. Prepare the click reaction cocktail immediately before use.
  a. Add the reagents in the order recommended by the manufacturer.
  b. Mix gently after each addition.
  c. Protect the cocktail from light.
20. Remove the PBS completely from the samples.
  a. Immediately add 300 μL of Click-iT reaction cocktail to each 35 mm glass-bottom dish.
  b. Gently rock the dish to ensure that the entire imaging area is evenly covered.
21. Incubate the samples for 30 min at 20°C–25°C, protected from light.
22. Remove the reaction cocktail and wash the samples three times with PBS for 5 min each on a shaker.

***Note:*** In the validated workflow described here, the clickable nucleoside analog was detected using the Click-iT EdU Imaging Kit with Alexa Fluor 647 azide. Alexa Fluor 647 is widely used for STORM because it provides robust photoswitching in reducing, oxygen-scavenging imaging buffers. Alternative fluorophore azides may show different blinking behavior and should be validated before use for single-molecule localization microscopy. For each 35 mm glass-bottom dish, 300 μL reaction cocktail was prepared by mixing 231 μL 1× reaction buffer, 6 μL CuSO_4_, 3 μL Alexa Fluor 647 azide, and 60 μL 1× buffer additive. The principle of EdU detection by azide–alkyne click chemistry is shown in Figure 2B. If a different clickable nucleoside analog, fluorophore azide, or click chemistry kit is used, prepare the reaction cocktail according to the manufacturer’s instructions and validate labeling efficiency, background signal, spectral compatibility, and STORM blinking performance before formal imaging.

23. Remove the PBS completely from the samples. Denature DNA by adding 1 mL of 2 M HCl to each dish and incubating for 30 min at 37°C.

**CRITICAL:** Efficient DNA denaturation is required for reliable antibody-based detection of halogenated nucleoside analogs such as BrdU, IdU, or CldU. Insufficient denaturation can lead to weak staining, whereas excessive denaturation can damage nuclear morphology and impair subsequent imaging. The denaturation condition should be optimized when cell type, fixation condition, or sample density is changed.

**CRITICAL:** Handle 2 M HCl in a fume hood and avoid direct skin or eye contact.

24. Immediately remove the HCl solution and wash the samples three times with PBS.
25. Wash the samples again three times with PBS for 5 min on a shaker.
26. Prepare the primary antibody solution for the nucleoside analog to be detected.

***Note:*** For BrdU detection in the validated workflow, dilute mouse anti-BrdU antibody 1:50 in blocking buffer containing 5% BSA and 0.5% Triton X-100.

***Note:*** BSA was used as a species-independent blocking reagent and provided a suitable signal-to-background ratio in fixed HeLa cells. Normal serum from the host species of the secondary antibody may be used as an alternative if nonspecific background is high, following validation.

27. To save antibody amount, add 30 μL primary antibody solution to each dish. To ensure even distribution of the antibody across the imaging area while minimizing antibody consumption, cover the sample with an adhesive sample coverslip film (Figure 3A).

**Figure 3:**
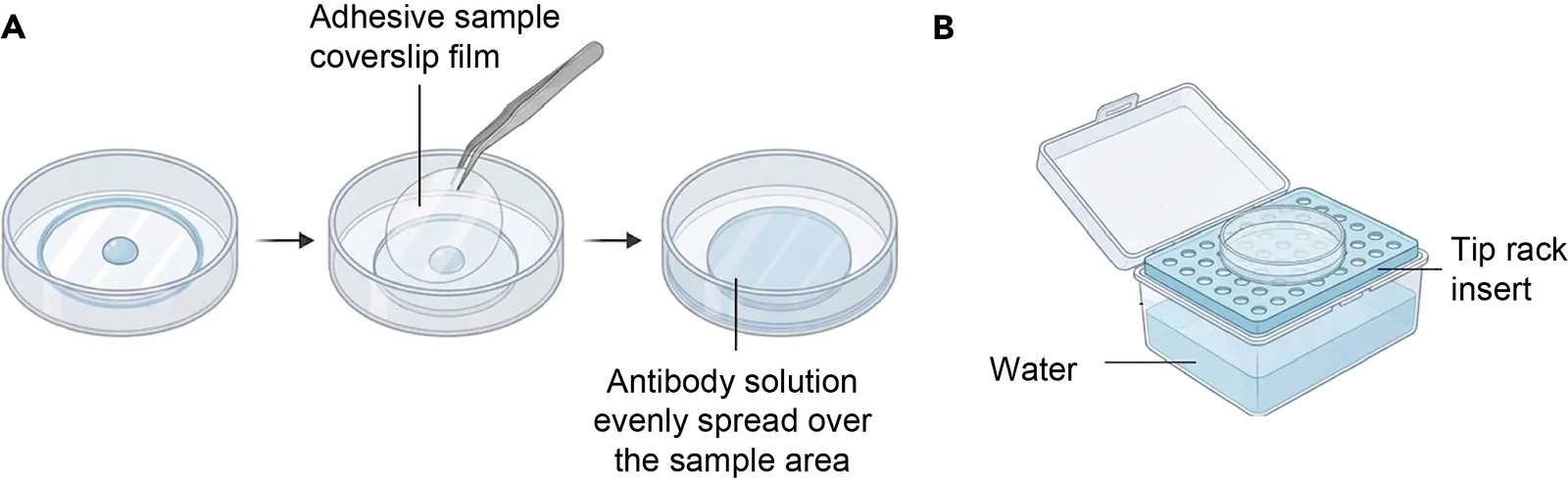
Setup for low-volume incubation on a glass-bottom dish (A) Schematic showing the addition of a small volume of solution to the center of the dish and placement of an adhesive sample coverslip film over the imaging area to ensure uniform coverage of the central glass-bottom region while minimizing reagent consumption. (B) Schematic of a homemade humidified chamber assembled from an empty pipette tip box. Water was added to the lower compartment, and the dish was placed on the tip rack insert during incubation.

***Note:*** This approach helps confine the antibody solution to the imaging area of the 35 mm glass-bottom dish.

28. Incubate the samples for 12–16 h at 4°C in a humidified chamber.

**CRITICAL:** To prevent sample drying, prepare the humidified chamber using an empty pipette tip box with water added to the lower compartment, and place the dishes on the upper rack. Keep the chamber closed and protect the samples from light throughout the incubation (Figure 3B).

***Note:*** For the anti-BrdU primary antibody, a 1 h incubation at 20°C–25°C was tested but produced weaker staining than incubation for 12–16 h at 4°C.

29. The next day, gently remove the adhesive sample coverslip film using clean tweezers. Wash the samples three times with PBS for 5 min each on a shaker.
30. Prepare the species-matched fluorescent secondary antibody solution for the nucleoside analog to be detected.

***Note:*** In the validated workflow described here, BrdU detection was performed using an in-house Cy3B-conjugated donkey anti-mouse IgG secondary antibody prepared in advance as described in the Preparation of dye-conjugated secondary antibody section and illustrated in Figure 1. The antibody was diluted 1:50 in blocking buffer containing 5% BSA and 0.5% Triton X-100, corresponding to a final concentration of approximately 10 μg/mL. The principle of BrdU detection by immunofluorescence is illustrated in Figure 2C. Commercially available secondary antibodies with similar spectral properties may also be used after validation of staining specificity, background signal and STORM blinking performance.

31. Add 30 μL secondary antibody solution to each dish. To ensure uniform antibody coverage of the imaging area while minimizing antibody consumption, cover the sample with an adhesive sample coverslip film.
32. Incubate the samples for 1 h at 20°C–25°C, protected from light.

***Note:*** Keep the samples protected from light during secondary antibody incubation and all subsequent steps to minimize photobleaching.

33. Gently remove the adhesive sample coverslip film using clean tweezers. Wash the samples three times with PBS for 5 min each on a horizontal shaker, at ∼1 Hz.
34. Post-fix the samples with 4% formaldehyde for 10 min at 20°C–25°C.
  ***Note:*** Post-fixation stabilizes antibody–antigen complexes and fluorescent labels at their target sites before STORM imaging.^16^
  a. Remove the formaldehyde solution.
  b. Wash the samples three times with PBS for 5 min each on a shaker.
35. Store the labeled samples in PBS at 4°C, protected from light, until bead incubation and fluorescence imaging.

**Pause point:** Labeled samples can typically be stored in PBS at 4°C, protected from light, for 1–2 days before imaging. Minimize storage time when possible to preserve signal quality.

***Optional:*** If longer storage is required, wash the samples five times with distilled water, remove the residual liquid completely, and allow the samples to air-dry naturally. Store the dried samples at 4°C, protected from light, until further use.

### Protein immunofluorescence labeling

**Timing: 1–2 days, including 12–16 h primary antibody incubation at 4°C**

This section describes immunofluorescence detection of a nuclear protein.

***Note:*** This protein-immunofluorescence module is presented as an alternative to dual nucleoside-analog labeling. In the validated workflow, EdU click chemistry is combined with immunofluorescence detection of a nuclear protein. Although not included in the example workflow, dual BrdU/protein immunofluorescence can also be performed.

Protein immunofluorescence can be performed on parallel samples or incorporated into the nucleoside analog-labeling workflow when antigen preservation is maintained and the fluorophores have adequate spectral separation. This module can be used to detect replication-associated proteins, chromatin regulators, nuclear structural proteins, DNA damage markers, etc. Because HCl denaturation for nucleoside analog detection can compromise some protein epitopes, each protein marker should be tested before being combined with acid-denaturation-based staining.

36. Protein immunofluorescence labeling.
  a. After click chemistry, prepare the primary antibody solution by diluting the selected primary antibody in blocking buffer containing 5% BSA and 0.5% Triton X-100.
  b. Add 30 μL primary antibody solution to each 35 mm glass-bottom dish and cover the imaging area with an adhesive sample coverslip film to ensure even antibody distribution while minimizing reagent consumption.
  c. Incubate the samples for 1 h at 20°C–25°C or 12–16 h at 4°C, protected from light.
    ***Note:*** Select the incubation condition according to the antibody and target protein. For 12–16 h incubation at 4°C, place the dishes in the humidified chamber described in step 28 to prevent evaporation of the low-volume antibody solution.
  d. Gently remove the adhesive sample coverslip film using clean tweezers and wash the samples three times with PBS for 5 min each on a shaker.
  e. Prepare the secondary antibody solution by diluting a species-matched fluorescent secondary antibody in blocking buffer containing 5% BSA and 0.5% Triton X-100.
  f. Add 30 μL secondary antibody solution to each dish.
    i. Cover the imaging area with an adhesive sample coverslip film.
    ii. Incubate the samples for 1 h at 20°C–25°C, protected from light.
  g. Remove the adhesive sample coverslip film and wash the samples three times with PBS for 5 min each on a shaker.
  h. Post-fix the samples with 4% formaldehyde for 10 min at 20°C–25°C, followed by three washes with PBS before storage or direct imaging.
    ***Note:*** For the CST PCNA antibody used here, a 10 min pre-treatment with cold methanol at −20°C can improve staining performance. After methanol treatment, wash the samples with PBS and proceed with antibody staining as described above.

### Prepare samples for dual-color 2D STORM imaging

**Timing: 1–2 h before imaging**

This section describes the addition of fiducial beads and the exchange of labeled samples into imaging buffer before dual-color 2D STORM acquisition.

***Note:*** Fiducial beads provide stable reference signals for lateral x-y drift correction and interchannel registration. The samples are then exchanged from PBS into freshly prepared STORM imaging buffer.

37. Before STORM imaging, examine the labeling quality of the samples using a conventional fluorescence microscope or the widefield mode of the STORM system. At this stage, PBS is sufficient for initial screening.
38. Select samples with intact nuclear morphology and clearly detectable signals in the intended imaging channels for subsequent STORM acquisition.

***Note:*** If nucleoside analog-labeled signals or protein immunofluorescence signals are too weak or highly heterogeneous across cells, re-optimize cell-cycle enrichment, pulse-labeling conditions, denaturation, antibody staining, or click chemistry before super-resolution imaging.

39. Prepare diluted solution of TetraSpeck microspheres listed in the key resources table.
  a. Centrifuge before use to remove bead aggregates.
  b. Dilute the bead stock solution to an appropriate working concentration (e.g., 1:1800; serial dilutions may be required) such that beads are evenly and broadly distributed across the field of view (FOV) without excessive crowding that could interfere with imaging of the target structures.
  c. Depending on the size of the glass-bottom area, a volume of about 70–300 μL is typically sufficient.
  d. Add the beads solution to samples and incubate for 30 min at 20°C–25°C to allow the beads to stably adhere to the glass surface.

***Note:*** The fluorescent beads are used for subsequent interchannel registration and lateral drift correction and need to be detectable in all channels used for imaging.

***Note:*** Gold nanoparticles can be used as alternative fiducial markers if they are compatible with the optical setup. They provide a continuous signal without photobleaching and may be advantageous for long acquisitions. Particle size and concentration should be optimized before use.

40. Wash three times with PBS to remove free or weakly bound beads. The bead distribution can then be checked under a conventional fluorescence microscope.

***Note:*** Ideally, 1–6 stationary beads should be visible within a single FOV of approximately 60 × 60 μm. Too few beads make it difficult to identify bead-containing FOVs and hinder subsequent drift correction and channel registration. Too many beads may interfere with target signal acquisition or complicate ROI selection. Optimize bead dilution before formal imaging.

41. Prepare the STORM working imaging buffer.
  a. Mix STIB, GLOX stock, and β-ME at a ratio of 100:1:1. For example, add 20 μL GLOX stock and 20 μL β-ME to 2 mL STIB.
  b. Mix gently, avoid generating bubbles, and use the buffer within 2 h.

***Note:*** STIB is stored at 4°C and can be taken out in advance to equilibrate to 20°C–25°C, thereby reducing drift caused by laser-induced warming during imaging. β-ME has a strong odor and strong reducing activity, so minimize its exposure to air and aspirate it from below the liquid surface whenever possible. After aliquoting GLOX, return it to the −20°C freezer as soon as possible to minimize loss of activity. After all components are mixed, the buffer can be left for 15–30 min to further deplete dissolved oxygen, because oxygen promotes photobleaching and degrades blinking quality. If too many fluorophores are simultaneously in the ON state, their single-molecule signals may overlap within individual frames.^9^ In this case, increase the β-ME proportion gradually and reassess the blinking density.

42. Remove the PBS from the sample and add freshly prepared STORM working imaging buffer.
  a. Do not dry the cell area while aspirating the liquid.
  b. Add enough imaging buffer to fully cover the sample region (for a standard glass-bottom dish, 2 mL is sufficient) and minimize bubble formation.
  c. If one sample requires prolonged acquisition, the dish can be sealed to minimize oxygen dissolution from the environment.
  d. Unused STORM working imaging buffer should not be stored for future use.

### Dual-color STORM imaging and acquisition

**Timing: 2–3 days**

This section describes dual-color STORM acquisition after sample preparation.

***Note:*** In the validated workflow described here, the 647 channel is used to detect short-pulse nucleoside analog-labeled replication-associated signals, whereas the 561 channel is used to detect longer-pulse nucleoside analog-labeled replication-associated signals. If different nucleoside analogs, antibodies, fluorophores, or protein markers are used, the acquisition workflow can be adapted to other channels or targets after validation.

***Note:*** In the associated study, the longer-pulse 561-channel (BrdU-Cy3B-labeled) signals were analyzed as longer-pulse signal structures, whereas the short-pulse 647-channel (EdU-Alexa Fluor 647-labeled) signals were interpreted as short-pulse signal clusters. These biological interpretations depend on synchronization strategy, pulse duration, labeling efficiency, and downstream image analysis.

43. Turn on the STORM system according to the local microscope standard operating procedure (SOP), such as the inverted microscope, motorized stage, EMCCD or sCMOS camera, 405 nm, 561 nm, and 647 nm lasers, laser attenuation and modulation devices, and near-infrared focus-lock system.
  a. Begin formal acquisition only after the lasers and microscope have stabilized.
  b. Settings for the home-made STORM platform are provided in Methods S1, section 1. Because hardware configurations differ among STORM platforms, the values are provided only as a reference and should be adjusted for the specific hardware and according to the resulting imaging performance.
44. Identify cells for imaging at low laser power. After the focal plane has been adjusted, engage focus lock and prepare for acquisition (see Methods S1, section 2 for details of the focus-lock system). Cells in the selected FOV should meet the following criteria:
  a. Signals are detectable in the intended imaging channels within the same target cell or nuclear region.
  b. Stable fluorescent beads are present in the FOV for subsequent registration and correction.
  c. Avoid FOVs with obvious abnormalities, such as nuclei cut off at the FOV boundary.

***Note:*** Use the near-infrared focus-lock system to monitor axial drift during acquisition. Engage focus lock after the focal plane is selected and confirm that the focus-lock signal remains stable throughout image acquisition. The focus-lock system monitors the displacement of the near-infrared reference signal and applies feedback to the objective position to maintain the focal plane.

45. Record widefield images in the 647 and 561 channels sequentially at low laser power as references.
  a. For each reference image, acquire 50–100 frames at a suitable exposure time, for example 30 ms, so that the frames can later be averaged to reduce noise.
  b. These images can be used for identification of cell and bead regions of interest (ROIs).
46. Acquire STORM data in the 647 channel.
  a. With the 405 nm laser turned off, illuminate the sample with a high-power 647 nm laser (≥10 kW/cm^2^ or as the maximum power density of the system) to turn off most fluorophores in the 647 channel into the dark state, so that the single-molecule blinking density becomes suitable and individual point spread functions (PSFs) no longer overlap.
  b. Adjust the 647 nm laser power to 1–3 kW/cm^2^ and begin acquisition of the STORM image stack.
    ***Note:*** The required number of frames depends on the desired final image quality: more frames yield more localizations but also require longer acquisition. In this example, 60,000 frames were collected at 30 ms exposure time (approximately 30 min per channel) and EM gain 100. For sCMOS cameras, EM gain is not available and is not required. Exposure time and laser power can be adjusted according to the final super-resolution reconstruction quality.
  c. Monitor the blinking-event density during acquisition. When the blinking-event density becomes insufficient, apply 405 nm illumination to reactivate fluorophores from the dark state.
    **CRITICAL:** Only weak 405 nm illumination is usually required (power density on the order of 10 W/cm^2^), so the power should be increased gradually from the minimum setting to avoid excessively high blinking density.
    ***Note:*** Under the imaging conditions used in this workflow, the blinking-event density typically became insufficient after approximately 10 min. A 405 nm laser is not required at the beginning of acquisition. If a 405 nm laser is unavailable, continue acquisition while the blinking-event density remains sufficient. The total acquisition time may need to be reduced because later frames can yield too few localization events. Fewer acquired localizations may reduce the quality of the reconstructed STORM image. For targets with different labeling densities or for other fluorophores, acquisition may be adequate without 405 nm illumination; this should be determined empirically for each experiment.
  d. Use a consistent naming convention and folder structure when saving data so that downstream processing scripts can recognize files in batch processing.
  e. Create an independent folder for each FOV and retain clear labels such as 647st or 561st in file names.
  f. Record the acquisition date, sample condition, FOV identifier, channel information, and related metadata.
47. Switch to 561 nm laser excitation and use the corresponding filter set while maintaining the same FOV and focus-lock state and acquire STORM data in the 561 channel. The remaining steps are the same as in Step 46.
  a. First use a high-power 561 nm laser to turn off most fluorophores into the dark state.
  b. Then acquire the 561-channel STORM image stack under lower laser power density.
  c. When the single-molecule blinking density decreases, use the 405 nm laser as needed.
  d. Use a similar saving method as 647 channel.

***Note:*** In the validated workflow described here, acquire the 647 channel before the 561 channel. Because fluorophore absorption spectra often include a short-wavelength absorption wing, 561 nm excitation can also excite fluorophores detected in the 647 channel, generating crosstalk and false-positive localizations between channels. In addition to a multi-channel bandpass emission filter, a single-channel bandpass emission filter can also be used to reduce crosstalk. If different fluorophores or channels are used, determine the acquisition order using single-labeled controls.

48. Verify that data were saved correctly, and record key features of the acquisition, such as the number of target cells or nuclei in the FOV, the corresponding ROIs, signal quality, bead distribution, imaging channel order, and any abnormalities observed during acquisition.
49. Move to a new FOV and repeat Steps 44–48 until sufficient data have been collected. If imaging exceeds 2 h or the blinking quality of fluorophores declines noticeably, replace the STORM working imaging buffer.

***Note:*** Do not exchange the buffer during acquisition of a single FOV, because buffer exchange can disturb focus stability, bead position, and channel registration.

50. After imaging of one sample is completed, turn off laser output, disengage focus lock, and switch to the next sample. Repeat Steps 37–49 for each new sample to complete data collection for different samples.
51. After data acquisition is complete, shut down each hardware component according to the microscope SOP.
52. If the samples need to be stored after imaging, gently wash them three times with deionized water and remove the residual liquid completely. Keep the dishes upside down at 4°C, protected from light. Under these conditions, dried samples can generally be stored for more than 3 months.

***Note:*** Approximately 50 min of preparation is required before each imaging session, and acquisition of each FOV takes about 1 h. The total number of FOVs acquired depends on the number of cells required for statistical analysis. For each sample condition, at least 15 FOVs are typically collected, with multiple analyzable cells present in each FOV when possible.

### Reconstruct STORM images and quantify replication-associated signal clusters

**Timing: 2–4 days**

This step describes how to reconstruct super-resolution images from raw dual-color STORM data and quantitatively characterize replication-associated signal clusters.

***Note:*** The core workflow includes ROI cropping, ThunderSTORM^13^-based single-molecule localization, bead-based drift correction, channel-specific processing of the 561 nm and 647 nm image stacks, SR-Tesseler segmentation, and cell-level statistical analysis. To ensure comparability across sample conditions, all datasets should be processed with the same parameter settings. The overall data-processing flow is shown in Figures 4 and 5.

53. Organize the raw data and determine the ROI cropping regions for each FOV.
  a. The folder for each FOV should contain consistently named files such as 647st∗.tif and 561st∗.tif, together with cell∗.roi and beads∗.roi files saved in ImageJ/Fiji, facilitating batch processing as the dataset grows. ROIs can be checked quickly using the widefield images.
    ***Note:*** The asterisk denotes a variable suffix. For example, files can be named 647st_001.tif, 647st_002.tif, 561st_001.tif, and 561st_002.tif.
  b. In Fiji,^12^ open the widefield reference image for the corresponding FOV.
    i. Define bead ROIs around well-isolated TetraSpeck microspheres.
    ii. Click “Add” in the ROI Manager, select the added ROI.
    iii. Choose More > Save to save it as a beads∗.roi file.
      ***Note:*** Representative bead ROIs are indicated by yellow dashed squares in Figure 4B. Each bead ROI should contain one isolated bead and a small surrounding margin.
  c. Define one cell ROI for each analyzable nucleus.
    i. Include one complete nucleus and a small surrounding margin.
    ii. Exclude neighboring nuclei and fiducial beads.
    iii. Click “Add” in the ROI Manager to add the cell ROI.
    iv. Select the added ROI and choose More > Save to save it as a cell∗.roi file.
      ***Note:*** The white dashed rectangle in Figure 4E indicates a representative cell ROI.
  d. Use the saved cell∗.roi and beads∗.roi files for ROI cropping in Step 54. The following STORM data processing flow corresponds to Figure 4A.
54. Crop cell and bead ROIs and concatenate the corresponding 647- and 561-channel stacks in Fiji.
  a. Open the 647-channel raw STORM stack for one FOV in Fiji. Open the ROI Manager through Analyze > Tools > ROI Manager. Load the corresponding cell∗.roi file. Select one cell ROI and crop the stack using Image > Crop.
  b. Open the matching 561-channel raw STORM stack. Load the same cell∗.roi file and crop the stack using the same ROI coordinates.
  c. Concatenate the cropped 647- and 561-channel stacks using Image > Stacks > Tools > Concatenate. Keep the 647-channel frames first and the 561-channel frames second. Save the concatenated stack as one BigTIFF file for each cell ROI.
  d. Repeat steps a–c for each bead ROI in the beads∗.roi file. Save each concatenated bead stack separately for drift-correction analysis.
55. Process bead ROIs in the ThunderSTORM^13^ plugin in Fiji and generate the drift-correction file. Representative parameter configuration interface of ThunderSTORM is shown in Figure 4D.
  a. Detailed instructions are available in ThunderSTORM documentation; representative operation screenshots are also provided in Methods S2.
  b. Operation manual:
    i. Import the data by “File > Open” or “File > Import > TIFF Virtual Stack”.
    ii. Configure the camera-specific parameters by selecting “ Plugins > ThunderSTORM > Camera setup”.
    iii. Then select “Plugins > ThunderSTORM > Run analysis” to open the “Run analysis” panel. After configuring the analysis parameters, click “OK” at the bottom of the panel to perform 2D localization analysis.
  c. Adjust the parameters according to the imaging system and data characteristics.
    i. The “Camera setup” section contains hardware-specific parameters such as pixel size, offset, and EM gain.
    ii. In the “Run analysis” panel, the peak intensity threshold is used for initial detection and screening of single-molecule signals.
    iii. In the “Sub-pixel localization” section, the fitting radius and initial sigma should be set according to the PSF size and the effective pixel size. A larger fitting radius (e.g., 5) improves fitting accuracy but increases computation time and requires greater inter-PSF spacing.
  d. After processing is complete, the “ThunderSTORM: results” window will appear.
    i. In the “Drift correction” tab, select “Fiducial markers”, select “Save to file”, specify the output location and filename.
    ii. Click “Apply” to calculate the x-y drift-correction file from the beads. This generates a “∗_drift.json” file that records the estimated drift trajectory.

***Note:*** Processing time depends on CPU performance and the number of detected events. A JSON file is a plain-text file that stores structured data. In this workflow, the “∗_drift.json” file stores the estimated x-y drift trajectory and is applied to the cell ROI data from the same FOV. This file is used for drift correction and alignment of the subsequent STORM data. Depending on the computer configuration, this step may require several tens of minutes.

56. Use ThunderSTORM to localize single molecules in nuclei from each FOV and apply the JSON file generated in the previous step for drift correction.
  a. Open the concatenated STORM data from Step 54 in Fiji and perform localization analysis.
    i. Import the data as Step 55 b (i). Use the same camera parameters as in the previous step because the data were acquired with the same camera.
    ii. Run analysis as Step 55 b (iii).
      ***Note:*** The analysis parameters, such as the peak intensity threshold and fitting radius, may require fine-tuning, as the PSF of fluorescent beads differs from that of single dye molecules in brightness and density.
  b. After localization, the “ThunderSTORM: results” window will appear.
    i. In the Drift correction tab, select “Load from file”, specify the JSON drift-correction file generated in Step 55 d(ii) under “Load file path”, and click “Apply” to perform x-y drift correction.
    ii. When correction is complete, click “Export” to save the drift-corrected localization data, for example as “∗_after_correction.csv”. This file serves as the input for subsequent two-color channel separation.
57. Split the merged localization results back into the 647 and 561 channels.
  a. In the “ThunderSTORM: results” window, switch to the “Filter” tab.
  b. Enter “frame < 60001″ or “frame > 60000″ in the “Filter” field, because 60,000 frames were acquired per channel in this example, and click “Apply” to separate the data into the 647- and 561-channel localization results.
  c. After applying each filter, click “Export” in the lower-right corner and save the corresponding output with an appropriate filename.
58. Visualize the final localization results.
  a. Import the CSV files into the ThunderSTORM plugin by selecting “Plugins > ThunderSTORM > Import results” in Fiji to render the super-resolution images.
  b. Click “Visualization” in the lower-right corner, adjust parameters such as the rendering method and magnification in the Visualization module to optimize the rendered output, and click “OK” to generate the rendered image.
59. Use SR-Tesseler^14^ to perform automated segmentation and quantitative analysis of the single-channel localization results.
  a. The processing flow is shown in Figure 5A and representative interfaces of SR-Tesseler are shown in Figures 5B–5E.
  b. Detailed instructions are available in the SR-Tesseler documentation; representative operation screenshots are also provided in Methods S2.
  c. Operation manual:
    i. In “SR-Tesseler: Viewer”, import the data (CSV localization results) from the upper left of the Viewer. Zoom out with the mouse wheel to facilitate visualization. In “SR-Tesseler: Controls” panel, check “Log scale” in the “Filters” tab.
    ii. Then, switch to “Voronoi diagram” tab and click “Create polygons” under “Voronoi construction” sub tab.
    iii. Next, switch to the “Objects” sub tab, set the relevant parameters, and click “Set density factor” and “Create objects” in sequence.
  d. In this example, the 561 channel corresponds to longer-pulse replication-associated signals.
    ***Note:*** Processing localization results yields metrics such as the signal cluster number, cluster area, and cluster size.
    During segmentation (Create objects), parameters can be guided by prior biological knowledge and inspection of representative reconstructions. For example, a minimum-area threshold (>7,500 nm^2^) was applied to exclude small fragments, and a density factor of 0.5 was used based on empirical testing of representative images.
    The “cut distance” parameter was not used because density-based segmentation provided satisfactory results for this dataset. However, it may be used if it provides better separation of adjacent clusters.
  e. In this example, the 647 channel corresponds to short-pulse replication-associated signals. The data can be imported and processed in the same way.
    ***Note:*** The resulting objects provide the quantitative basis for compact replication signal clusters. A minimum-area threshold may be unnecessary; instead, a higher local density factor is more appropriate. For example, a density factor of 5 provided a reasonable balance between compact structures and over-fragmentation.
  f. For each cell, export the object-level measurements from the 561-channel and 647-channel segmentation results.
    ***Note:*** These measurements include object number, area, size, and localization density and are used for subsequent statistical analysis.
    Parameters such as the density factor and minimum area in SR-Tesseler should be fixed and then applied consistently throughout downstream analysis.
60. Perform cell-level aggregation and statistical analysis of the SR-Tesseler outputs.
  a. For each cell or ROI, aggregate SR-Tesseler outputs to quantify signal cluster number, cluster density, cluster size, signal area, and spatial distribution for each imaging channel.
  b. When two labeled signal populations are analyzed together, calculate inter-channel spatial relationship metrics, such as nearest-neighbor distance, overlap fraction, centroid-to-centroid distance, or the number of compact signal clusters associated with each larger signal structure.
  c. Export both object-level and cell-level measurements for downstream statistical analysis. Apply the same ROI selection criteria, localization filtering, segmentation parameters, and statistical workflow to all sample conditions.

***Note:*** Processing time depends strongly on computer performance, the total number of FOVs, the number and size of ROIs, and the total number of frames. For a typical single FOV (60 × 60 μm) containing three nuclear ROIs and two channels with 60,000 frames per channel, processing requires about 2 h on an Intel i5-12600KF CPU.

**Figure 4:**
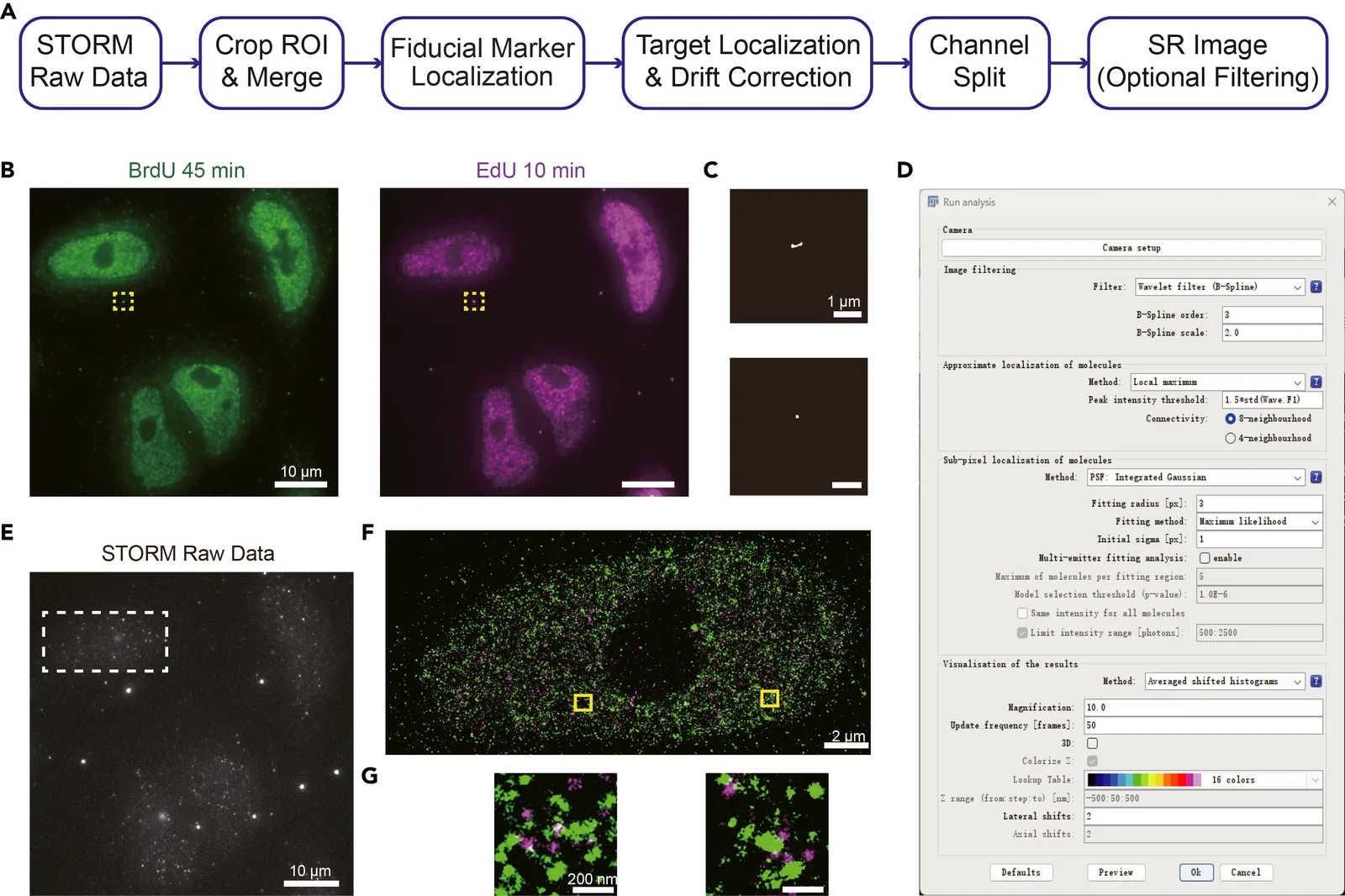
Schematic overview of the ThunderSTORM processing workflow (A) Workflow for dual-color STORM data processing corresponding to Steps 53–58. Optional localization filtering may be applied, depending on the quality-control criteria used. (B) Representative conventional fluorescence microscopy images of a complete FOV showing longer-pulse and short-pulse replication-associated signals in the 561 and 647 channels, respectively. Yellow dashed boxes indicate bead ROIs containing fiducial beads used for drift correction and channel registration, corresponding to the localization results shown in panel C. (C) Localization results of fiducial beads before and after drift correction. The upper panel shows bead localizations before correction, and the lower panel shows the corrected result. The signals shown correspond to reconstructed fiducial-bead localizations and were not subjected to segmentation. (D) ThunderSTORM interface. These parameters should be configured before they are finalized for batch processing. (E) Representative single-frame raw STORM image. The white dashed box marks the nuclear region corresponding to the reconstructed super-resolution image shown in panel F. (F) Representative dual-color STORM reconstruction. Green indicates the 561 channel (BrdU), and magenta indicates the 647 channel (EdU). (G) Magnified views of the yellow solid boxes in panel F, showing the spatial relationship between compact short-pulse signal clusters and larger longer-pulse signal structures. Scale bars: B and E, 10 μm; C, 1 μm; F, 2 μm; G, 200 nm.

**Figure 5:**
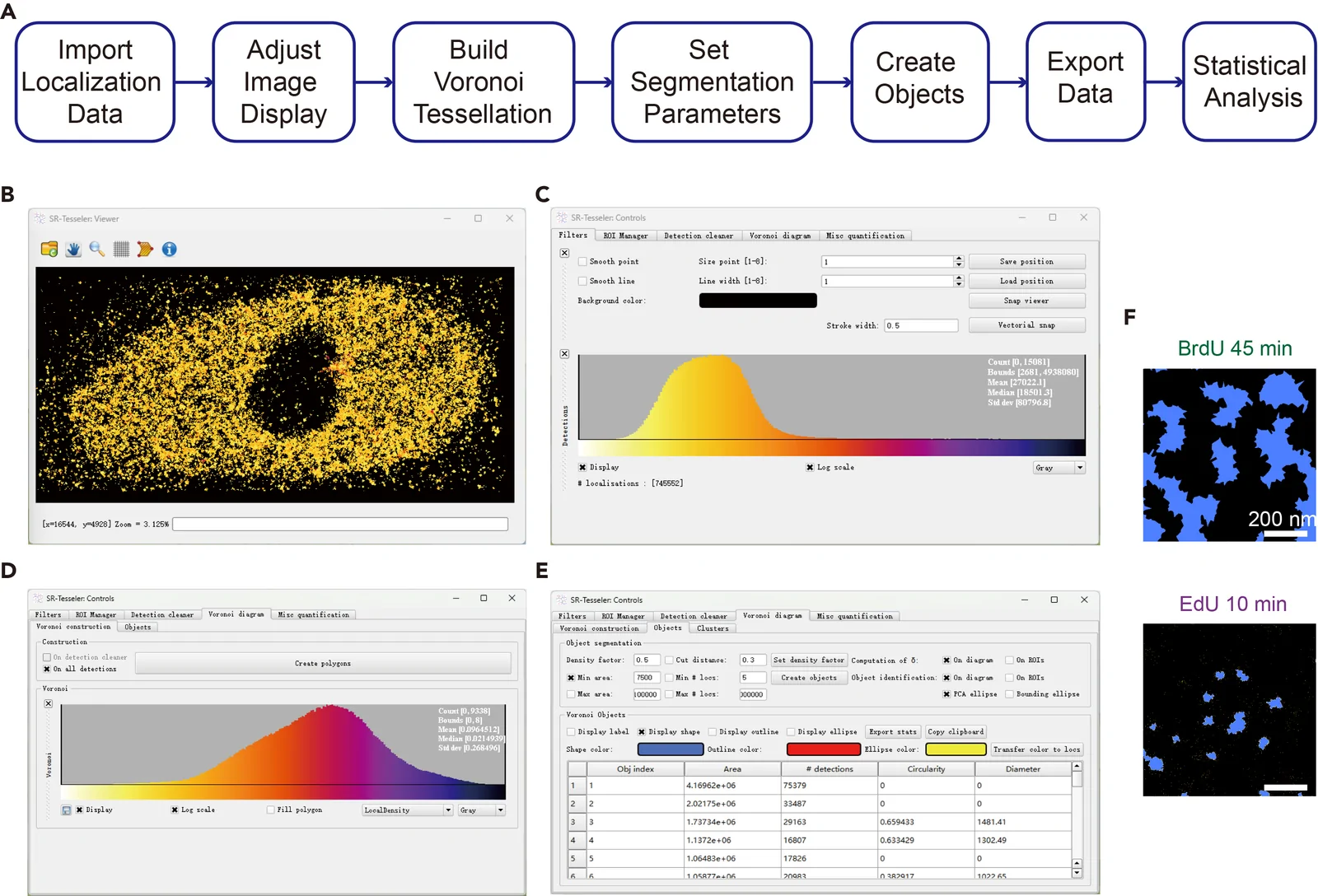
Schematic overview of the SR-Tesseler processing workflow (A) Workflow for segmentation and quantitative analysis of STORM localization data in SR-Tesseler. (B–E) SR-Tesseler interface. The panels show data import, display adjustment using log scale, creation of Voronoi polygons, and parameter-based object segmentation. (F) Representative segmentation of longer-pulse and short-pulse replication-associated signal clusters in the 561 (upper) and 647 (lower) channels, respectively. Replication domains in the 561 channel appear as relatively large spatial structures, whereas early replication sites in the 647 channel appear as compact punctate structures. Scale bars: 200 nm.

## Expected outcomes

Successful execution of this protocol should yield cell-cycle-enriched samples with nucleoside analog-labeled replication-associated signals. Before super-resolution imaging, conventional fluorescence inspection should reveal clearly detectable signals in the intended imaging channels within the same nucleus, with preserved nuclear morphology and low nonspecific background (Figure 4B). When two replication labeling windows are used, the two signal populations may differ in intensity, size, density, or spatial distribution depending on the synchronization strategy and pulse duration. Fluorescent beads added before STORM imaging should appear as bright stationary puncta in the FOV. The bead localizations from the yellow dashed boxes in Figure 4B are used to estimate lateral x-y drift and to register the 561- and 647-nm channels.

A representative single raw STORM acquisition frame is shown in Figure 4E. Bead-based drift correction should improve localization stability and channel alignment, as illustrated in Figure 4C. Reconstructed drift-corrected dual-color STORM images should reveal discrete replication-associated signal clusters in both imaging channels, allowing the spatial relationship between the two labeled signal populations to be evaluated at the nanoscale (Figures 4F and 4G). The STORM images before and after x-y drift correction are shown in Figure S3 in Methods S1.

Representative SR-Tesseler segmentation results are shown in Figure 5F. The larger blue objects in the upper panel represent larger, longer-pulse signal structures (561 channel), whereas the smaller, compact blue puncta in the lower panel represent short-pulse signal clusters (647 channel).

In addition to reconstructed images and segmentation overlays, the completed analysis workflow generates multiple output files, including localization files, object-level measurements, per-cell quantitative summaries, and statistical outputs. These files can be used for downstream analysis, quality control, troubleshooting, or re-analysis with alternative segmentation criteria.

## Limitations

This protocol depends heavily on robust and reproducible cell-cycle enrichment. Because replication-associated signals are captured through synchronization, release, and pulse labeling, small differences in cell density, washing quality, release timing, or pulse duration can substantially alter nucleoside analog incorporation and increase cell-to-cell heterogeneity. Therefore, all sample conditions should be processed in parallel under tightly matched culture, synchronization, labeling, and staining conditions. When the workflow is adapted to other cell types or biological questions, seeding density, synchronization duration, release timing, pulse-labeling conditions, and detection methods should be re-optimized.

A second limitation is the strong dependence of the assay on a well-optimized dual-color STORM imaging platform. This requirement reduces immediate transferability to laboratories without stable high-power lasers, sensitive single-molecule detectors, and experience with STORM acquisition. Although the upstream labeling and staining steps can be reproduced more broadly, the full quantitative output depends on successful nanoscale imaging and therefore remains instrument intensive.

This protocol is also limited by the sensitivity of STORM reconstruction and downstream segmentation to data quality and analysis parameters. The final measurements depend on fluorophore blinking behavior, imaging-buffer quality, localization density, signal-to-background ratio, acquisition stability, and accurate channel alignment. In addition, downstream quantification is not entirely parameter free. Localization filtering, reconstruction settings, and clustering thresholds in ThunderSTORM and SR-Tesseler influence the measured number, diameter, density, and spatial assignment of labeled signal clusters.

The present workflow is designed for two-dimensional STORM imaging and x-y localization analysis. In principle, this approach could be extended to three-dimensional imaging using astigmatism-based localization. Such an extension may provide additional information on the spatial organization of replication-associated DNA synthesis. However, it would require additional optical calibration, z-position calibration, and more complex data analysis than described here.

Manual selection of cell and bead ROIs remains a potential source of operator-dependent variability. Although consistent ROI criteria and identical localization and segmentation parameters are applied to all samples, variability introduced during manual ROI selection cannot be completely excluded. Future implementations may incorporate semi-automated nuclear segmentation, standardized image-based screening criteria, and inter-operator validation to improve reproducibility.

Finally, this protocol provides a fixed-cell, endpoint, and largely two-dimensional view of replication-associated DNA synthesis rather than a direct live-cell measurement of replication dynamics. Live-cell imaging of fluorescently tagged replication proteins provides a complementary approach. GFP-PCNA imaging can be used to monitor replication-foci dynamics,^17^ whereas single-particle tracking PALM of mEos2-PCNA can characterize PCNA mobility at the single-molecule level.^18^ However, these approaches report the behavior of replication proteins indirectly. They do not directly label newly synthesized DNA, as achieved by nucleoside analog labeling in the present fixed-cell protocol. The assay can resolve spatial organization and inter-channel relationships between labeled signal populations at high resolution, but it cannot directly capture the temporal sequence of origin licensing, origin firing, fork progression, or local chromatin remodeling in real time. Because representative FOVs and analyzable nuclei must be selected for imaging and reconstruction, the workflow remains relatively low throughput and may be affected by sampling bias if image-selection criteria are not predefined and applied consistently.

## Troubleshooting

### Problem 1

Cells fail to display the expected labeling pattern for the selected cell-cycle window, and nucleoside analog-labeled signals appear highly heterogeneous across nuclei (Steps 1–15).

### Potential solution

This problem is most commonly caused by differences in cell density, incomplete synchronization, residual synchronization drugs after washout, or inaccurate release timing. Ensure that cells are evenly seeded and reach approximately 20%–30% confluence before synchronization. Avoid overconfluent cultures, as high density can reduce synchronization efficiency and increase cell-to-cell heterogeneity.

In practice, absent or extremely weak nucleoside analog labeling often indicates that the synchronization drug was not washed out thoroughly enough. During each wash after thymidine or aphidicolin treatment, gently add pre-warmed PBS, swirl the dish thoroughly to ensure complete exchange of medium across the glass area, and remove the wash completely before adding fresh medium. Residual aphidicolin can strongly suppress S-phase entry and result in weak or absent nucleotide incorporation. In addition, release and pulse-labeling times should be strictly controlled and kept identical across all samples. If the labeling pattern remains heterogeneous, re-optimize the initial seeding density, synchronization duration, release timing, and pulse-labeling conditions for the specific cell batch or cell type used.

### Problem 2

Nucleoside analog-labeled signals are absent or very weak even though the synchronization and release steps have been confirmed to work properly (Steps 1–12 and 16–35).

### Potential solution

If synchronization quality, washing, and release timing have already been verified, weak or absent nucleoside analog-labeled signal is more likely caused by problems with the labeling or detection reagents. First check whether the nucleoside analog working solutions were prepared correctly, stored properly, and used within an appropriate time frame. Repeated freeze-thaw cycles, prolonged storage, or incorrect dilution may reduce labeling efficiency.

If click-based detection is weak, prepare the click reaction mixture freshly immediately before use and check each component carefully, especially the fluorescent azide, copper reagent, and reaction buffer. If antibody-based detection of halogenated nucleoside analogs is weak, verify that HCl denaturation is sufficient to expose the incorporated analogs and that the primary and secondary antibodies are active and used under optimized conditions. When necessary, test the click reaction reagents and antibody staining reagents separately using a positive-control sample that previously gave reliable signal.

### Problem 3

Nucleoside analog fluorescence appears uneven across the conventional fluorescence reference image, with strong signal in some regions and weak or absent signal in others (Steps 16–35).

### Potential solution

Uneven staining usually results from incomplete coverage of the glass surface by the reaction solution, sample drying during incubation, or inconsistent denaturation and antibody exposure. Make sure that all solutions fully cover the imaging area and that the dish remains level throughout incubation. When small staining volumes are used, avoid evaporation by keeping samples in a humidified chamber. During HCl denaturation, ensure that the solution is evenly distributed across the sample and that the incubation time is consistent between dishes. During antibody incubation, gently dispense the solution so that it spreads uniformly over the sample instead of remaining localized in one area. Also avoid allowing the sample to dry during washing or solution exchange, as even brief drying can create severe local staining artifacts.

Non-uniform illumination across the FOV may also cause spatial variability in signal intensity or localization density. This can be assessed by imaging a homogeneous fluorescent sample, such as a fluorescent calibration slide, under the same optical configuration.

### Problem 4

Background fluorescence is high, making it difficult to identify nucleoside analog-labeled or protein immunofluorescence structures under conventional fluorescence microscopy (Steps 16–35).

### Potential solution

High background usually reflects insufficient blocking or washing, excessive antibody concentration, residual fluorescent reagents, or sample deterioration during storage. Increase the blocking time if needed and verify that blocking buffer and antibody dilution buffer are freshly prepared. Reduce the concentration of the primary or secondary antibody if the target signal remains detectable while the background remains high. Extend washing time and perform washes more gently but more thoroughly, especially after click chemistry and secondary antibody incubation. If samples are not imaged immediately, store them under appropriate conditions and avoid prolonged storage before STORM acquisition, as background and nonspecific fluorescence may increase over time.

### Problem 5

Single-molecule blinking is poor during STORM acquisition, resulting in low signal-to-background ratio, insufficient localization density, or blurred reconstruction (Steps 37–52).

### Potential solution

Poor STORM image quality is often caused by suboptimal fluorophore performance, degraded imaging buffer, weak initial staining, or unstable imaging conditions. Prepare the STORM imaging buffer fresh on the day of use and begin STORM imaging within 15–30 min after preparation of the working imaging buffer. Also verify that the STIB is adjusted to pH 8.0 and that the reducing agent and oxygen-scavenging system are suitable for the fluorophores being used. In this workflow, β-ME and GLOX were used to support fluorophore switching. If alternative blinking-inducing compounds are used, their identity and concentration should be optimized together with the buffer pH. Before starting STORM acquisition, confirm under conventional fluorescence that the intended labeled signals are clearly detectable and that the selected cell or nucleus shows the expected signal pattern for the chosen labeling window. Optimize the excitation power, exposure time, and acquisition length so that fluorophores enter a suitable blinking regime without causing photobleaching. Also confirm that the microscope is in stable focus and that mechanical or thermal drift is minimized during acquisition. If signal remains weak, re-evaluate upstream labeling and staining quality before attempting to optimize the imaging settings further.

### Problem 6

Spectral crosstalk is observed between the 561- and 647-nm channels, making it difficult to distinguish the two labeled signal populations (Steps 39–47).

### Potential solution

Spectral crosstalk may result from insufficient spectral separation between the fluorophores or inappropriate emission filters and acquisition settings. Confirm that the selected filters and acquisition settings minimize bleed-through between the fluorophores, such as AF647 and Cy3B in the validated workflow. Image single-labeled control samples to determine whether fluorescence from one fluorophore is detected in the other channel. Adjust the optical filters or acquisition settings if substantial bleed-through is detected.

### Problem 7

Poor spatial registration is observed between the 561- and 647-nm channels, making the spatial relationship between the two labeled signal populations unreliable (Steps 53–58).

### Potential solution

Poor channel registration may result from chromatic aberration, optical misalignment, or inaccurate estimation of channel displacement. Insufficient or unevenly distributed fiducial beads do not themselves cause channel misregistration, but they reduce the accuracy of estimating and correcting channel displacement. Use fiducial beads detected in both channels to estimate and correct the spatial displacement. Ensure that multiple well-isolated beads are available for correction. Apply the same registration procedure to all samples.

### Problem 8

SR-Tesseler segmentation does not match visual inspection of the reconstructed images, leading to obvious over-segmentation or under-segmentation of labeled signal clusters (Steps 53–60).

### Potential solution

Segmentation errors are often caused by poor raw image quality, inconsistent localization filtering, or inappropriate density thresholds. Before changing segmentation parameters, first verify that the reconstructed images are based on good-quality localization data with sufficient point density and accurate drift correction. Then apply the same localization filtering and SR-Tesseler analysis pipeline to all samples. Do not adjust thresholds separately for different conditions unless there is a clearly justified and documented reason. If the segmentation result is unstable, inspect several representative nuclei and determine whether the density factor is too low, causing merged structures, or too high, causing fragmentation of a single structure into multiple clusters. The Cut distance parameter should also be evaluated because it controls the spatial connectivity of localizations. Depending on the selected value, it may contribute to merging or fragmentation of neighboring clusters. Quantitative comparisons should only be performed after identical analysis parameters produce segmentation results that are visually consistent with the reconstructed images across all sample conditions.

### Problem 9

Too few cells meet the quality criteria for downstream analysis, leading to poor reproducibility or insufficient sample size (Step 60).

### Potential solution

This problem is typically caused by a combination of suboptimal seeding density, cell loss during repeated washing and staining, low synchronization quality, and the inherently low-throughput nature of STORM imaging. To improve yield, optimize the initial seeding density so that cells remain well separated but abundant enough after synchronization and staining. Handle samples gently during all washing steps to reduce detachment. Establish predefined image-selection criteria, such as nuclear morphology, expected labeling pattern for the selected cell-cycle window, signal quality, bead distribution, registration quality, and absence of obvious artifacts, and apply these criteria consistently across all sample conditions. In addition, acquire data from multiple independent experiments rather than relying on a single imaging session, as this improves robustness and reduces sampling bias.

## Resource availability

### Lead contact

Further information and requests for resources and reagents should be directed to and will be fulfilled by the lead contact, Qian Peter Su (qian.su@uts.edu.au).

### Technical contact

Technical questions on executing this protocol should be directed to and will be answered by the technical contacts, Zhiguang Xiao (xiaozhiguang@stu.pku.edu.cn) and Xiaodong Guan (guanxd@stu.pku.edu.cn).

### Materials availability

All unique/stable reagents generated are available from the lead contact without restriction.

### Data and code availability

Any additional information required to reanalyze the data reported in this paper is available from the lead contact upon request.

## Acknowledgments

We gratefully acknowledge Dr Huiwen Hao from the Standard Imaging (Beijing) Biotechnology Co., Ltd. for sample preparation, technical support, and helpful discussion. This work was supported by the 10.13039/501100001809National Natural Science Foundation of China (22127804 to Y.S.), the 10.13039/501100012166National Key R&D Program of China (2022YFA1303103 and 2022YFA3401100 to Y.S.), the Australian National Health and Medical Research Council (Emerging Leadership APP1177374 and APP2041674 to Q.P.S.), and the 10.13039/501100000985NSW Cardiovascular Research Capacity Program Senior and Early-Mid Career Researcher Grant of Australia to Q.P.S.

## Author contributions

Z.X. developed and optimized the protocol. X.G. contributed to STORM system optimization and technical validation. M.Z., Y.S., and Q.P.S. supervised protocol development. Z.X., X.G., M.Z., and Q.P.S. co-wrote and revised the manuscript.

## Declaration of interests

The authors declare no competing interests.
