# Supplementary material for: Protocol for quantifying chromatin DNA replication by multiplex labeling and single-molecule localization microscopy
Source: STAR Protoc. 2026 Oct 7;7(4):104882. doi: 10.1016/j.xpro.2026.104882 (PMC13676695; doi:10.1016/j.xpro.2026.104882)
Supplement: Supplementary file 1 [file mmc1.pdf]

Methods S1. STORM acquisition settings, focus-lock characterization, and lateral-drift correction, related to Steps 43-56 and Figure 4.

Contents:

1. System-specific acquisition parameters
2. Principle and stability analysis of the focus-lock system
3. 561-channel STORM super-resolution images before and after x-y drift correction

1. System-specific acquisition parameters

The overall magnification of the microscope system was 139× (achieved using a two-lens relay system), giving an effective EMCCD pixel size of 115 nm and a field of view (FOV) of approximately 59 × 59 μm^2.

The MPB lasers have high output power. Although the power is adjustable, the minimum set output is 100 mW because of a hardware limitation. An acousto-optic tunable filter (AOTF) was therefore used for secondary adjustment of the laser power delivered to the sample. The AOTF was controlled using custom C++ software and calibrated in advance with a power meter, allowing the software transmittance setting to be adjusted from 0.1% to 100%. The software setting must be multiplied by the maximum transmittance permitted by the AOTF to obtain the physical transmittance. In this system, the maximum AOTF transmittance was 94%; thus, a software setting of 50% corresponded to an actual transmittance of 50% × 94% = 47%. Unless otherwise stated, transmittance values below refer to the values entered in the control software.

For the 647 nm laser, the output power was set to 100 mW. For conventional fluorescence imaging, the AOTF transmittance was adjusted between 1% and 20% according to the fluorophore properties, exposure time, and desired analog-to-digital unit (ADU) intensity. For STORM imaging, the AOTF transmittance was set to 100%, corresponding to a power density of 1 kW/cm^2 at the sample.

For the 561 nm laser, the output power was set to 200 mW because a nominal setting of 100 mW produced an inaccurate actual output of approximately 167 mW. For conventional fluorescence imaging, the AOTF transmittance was adjusted between 1% and 10% according to the fluorophore properties, exposure time, and desired ADU intensity. For STORM imaging, the AOTF transmittance was set to 50%.

Because the 405 nm laser had relatively low power, its output intensity could be adjusted directly without passing through the AOTF. During STORM acquisition, if the density of fluorophores in the on state was too low, the 405 nm laser was activated and its power was increased gradually from 0.2 mW until the density of on-state fluorophores in the STORM images was appropriate. The required power was generally below 2 mW and did not exceed 10 mW.

The output power of the Thorlabs near-infrared focus-lock laser was controlled by adjusting the diode current using a Thorlabs LDC205C controller and a Thorlabs LDM9LP laser-diode mount. For the 8-bit infrared camera (Thorlabs DCC1545M), a diode-current setting of 21–30 mA was generally sufficient. However, the required current depends on the characteristics of the specific laser diode and may need to be increased.

A ZT405/488/561/647rpc multiband dichroic mirror was installed in the microscope body, and a ZET405/488/561/647m was used as the first fluorescence emission filter. During acquisition in the 561 or 647 channel, a second fluorescence emission filter (FF01-585/40-25 or ET690/70m, respectively) was placed outside the microscope body. Filter selection should be determined according to the excitation wavelength and the spectrum of the fluorophore used.

2. Principle and stability analysis of the focus-lock system

The axial piezo stage was a P-726 from Physik Instrumente. As illustrated in Figure S1A–C, changes in the axial sample position were monitored from the resulting displacement of the reflected infrared spots. The axial stage was then driven in the opposite direction to maintain a stable imaging focal plane. Splitting the infrared laser into two beams (Figure S1D) increased system sensitivity and reduced interference from fluctuations in the individual spots. The infrared-camera image therefore contained two spots (Figure S2A).

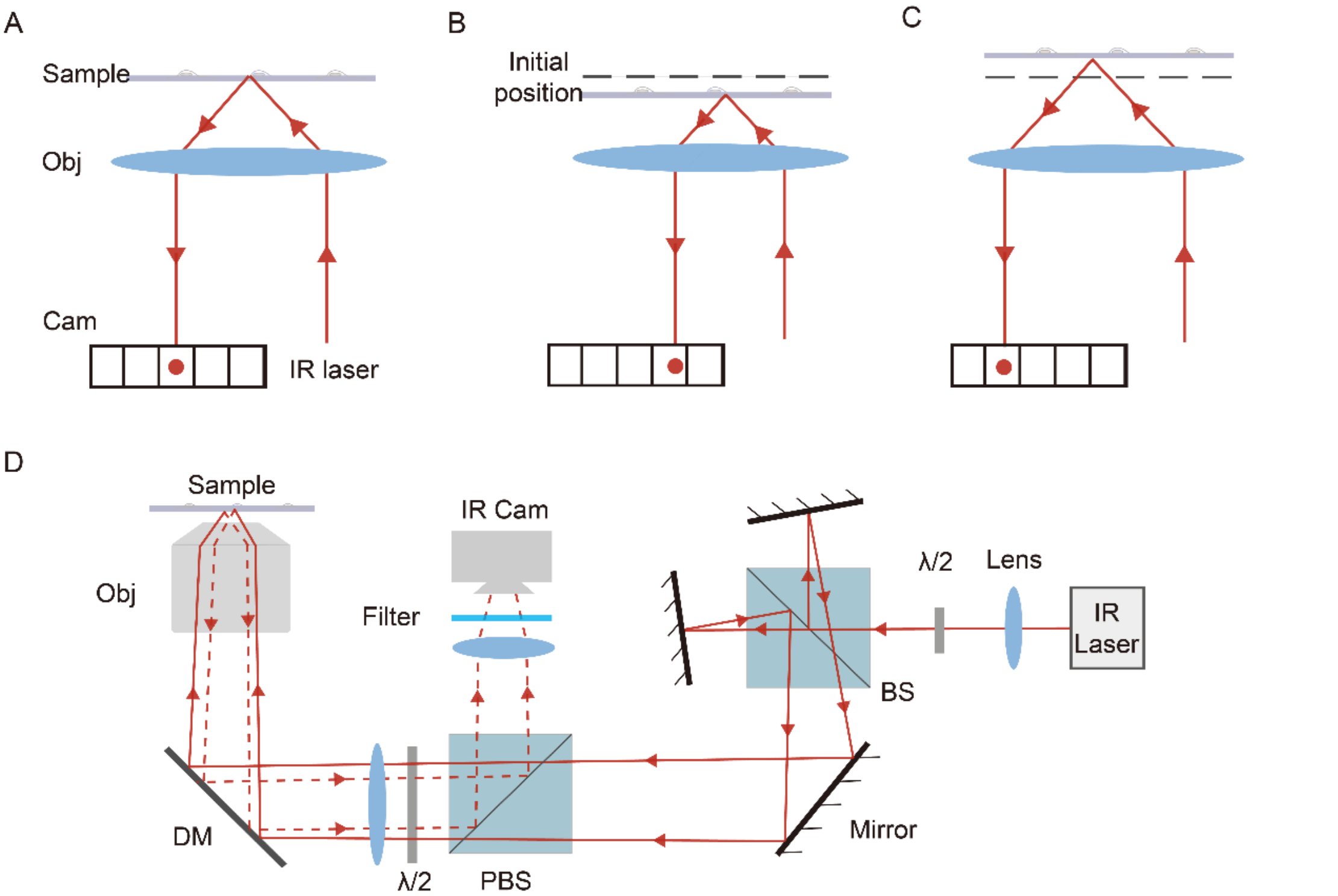

Figure S1. Principle of axial-drift detection and beam-splitting scheme for the focus-lock system, related to Step 44.

(A) When the sample is at the focal plane, the reflected infrared laser is directed to the center of the camera. The objective is shown schematically. Arrows indicate the direction of light propagation; repeated components are not labeled again in subsequent panels. (B) The dashed line indicates the initial position. When the sample moves downward, the reflected

infrared spot moves to the right on the camera. (C) When the sample moves upward from its initial position, the reflected infrared spot moves to the left. Adjusting the objective position with the axial stage to keep the infrared-spot positions constant maintains a stable focal plane. (D) Beam-splitting scheme for dual-beam focus locking. The dual-beam feedback system is more sensitive and stable than a single-beam system. A beam-splitter cube (BS) divides the laser into two beams, and the combination of a polarizing beam-splitter cube (PBS) and a half-wave plate (λ/2) effectively suppresses stray light in the camera image.

To evaluate focus-lock stability, a fluorescent-bead sample was imaged in two planes for 3,600 s (Figure S2B), followed by 3D localization. The axial positions of the beads in the FOV were averaged to obtain a mean axial-position trace. The standard deviation of the mean-position trace was then calculated over 60 s windows, with the initial axial position defined as the target (Figure S2C). The maximum peak-to-peak fluctuation in bead axial position was 10.9 nm; 3σ was 5.37 nm and 5σ was 8.9 nm. Statistically, only approximately one point in 1.7 million would be expected to fall outside the 5σ range. The sample could therefore be considered axially stable within approximately 10 nm.

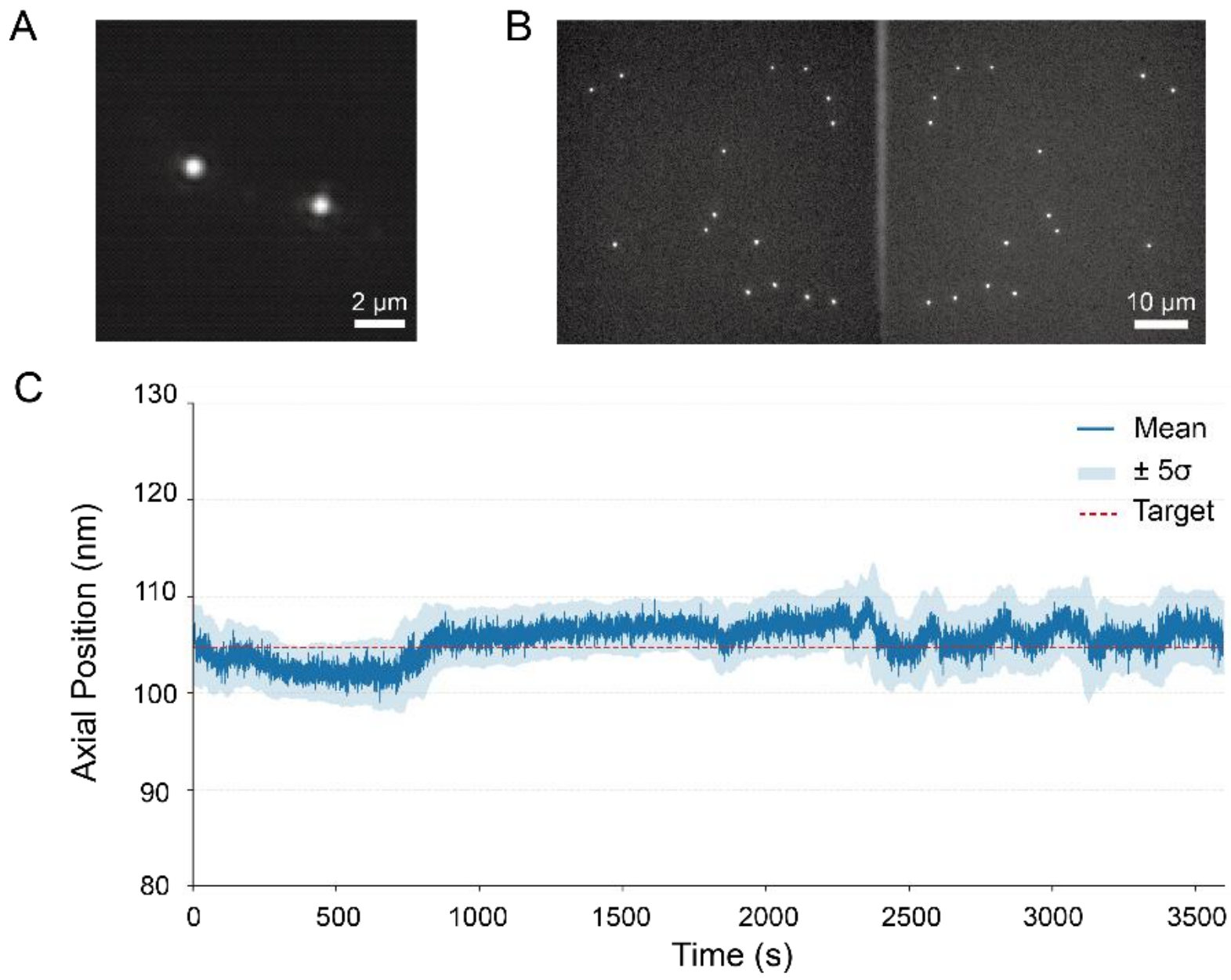

Figure S2. Imaging results and stability of the focus-lock system, related to Step 44.

(A) Representative infrared-camera image showing two spots. The centers of the two spots were localized, and negative feedback was used to keep the distance between them constant and stabilize the focal plane. (B) Biplane image of fluorescent beads. Axial localization analysis of fluorescent beads is used to evaluate focus-lock stability. (C) Fluctuation in fluorescent beads axial position over 3,600 s. The dark-blue line shows the mean axial position of beads in the FOV (Mean), and the light-blue region shows ±5 standard deviations (±5σ) of the bead axial positions over 60 s. The mean bead position fluctuated within 10 nm. Scale bars: A, 2 μm; B, 10 μm.

3. 561-channel STORM super-resolution images before and after x-y drift correction

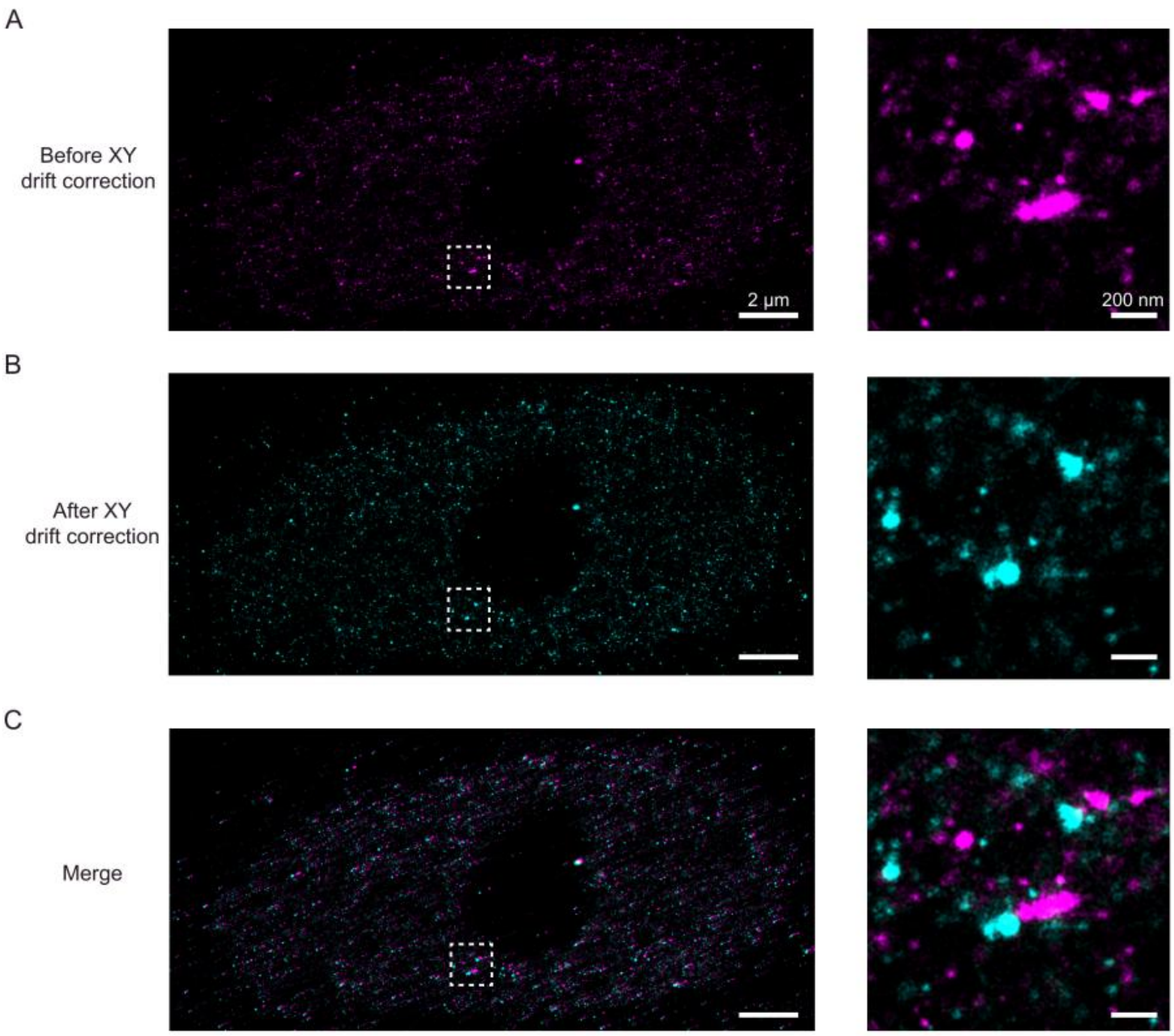

Figure S3. 561-channel STORM images before and after x-y drift correction, related to Step 56.

Super-resolution images of the 561 channel. (A) Before x-y drift correction. (B) After x-y drift correction. (C) Overlay of panels A and B, showing an evident displacement between the images before and after drift correction. The white dashed box highlights a region in which lateral drift correction restores localizations that had been elongated or erroneously separated into distinct points. On the right is an enlarged view of the dashed box area. Scale bar: 2 μm (left column), 200 nm (right column).
