# Supplementary material for: Protocol for quantifying chromatin DNA replication by multiplex labeling and single-molecule localization microscopy
Source: STAR Protoc. 2026 Oct 7;7(4):104882. doi: 10.1016/j.xpro.2026.104882 (PMC13676695; doi:10.1016/j.xpro.2026.104882)

Protocol for quantifying chromatin DNA replication by multiplex labeling and single-molecule localization microscopy

Methods S2. Illustrated instructions for ThunderSTORM reconstruction and SR-Tesseler segmentation, related to Steps 55–59 and Figures 4 and 5.

Contents:

ThunderSTORM operation manual

P3 – P8

SR-Tesseler operation manual

P10 – P14

ThunderSTORM operation manual

ThunderSTORM operation manual

version dev-2016-09-04-b1 includes biplane localization, stable version 1.3 is sufficient when only 2D localization is required.

(1) Import the data

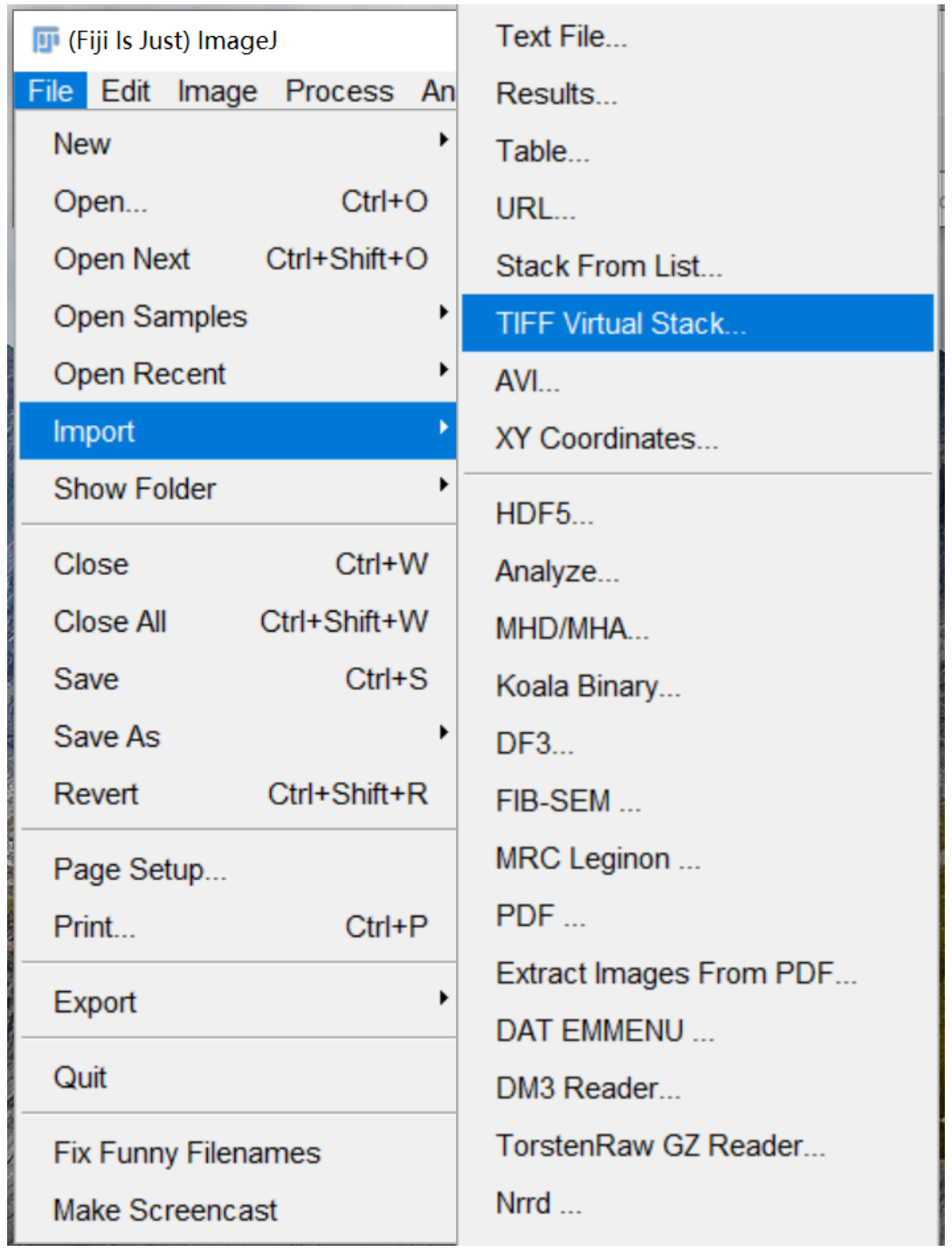

(2) Configure the camera parameters

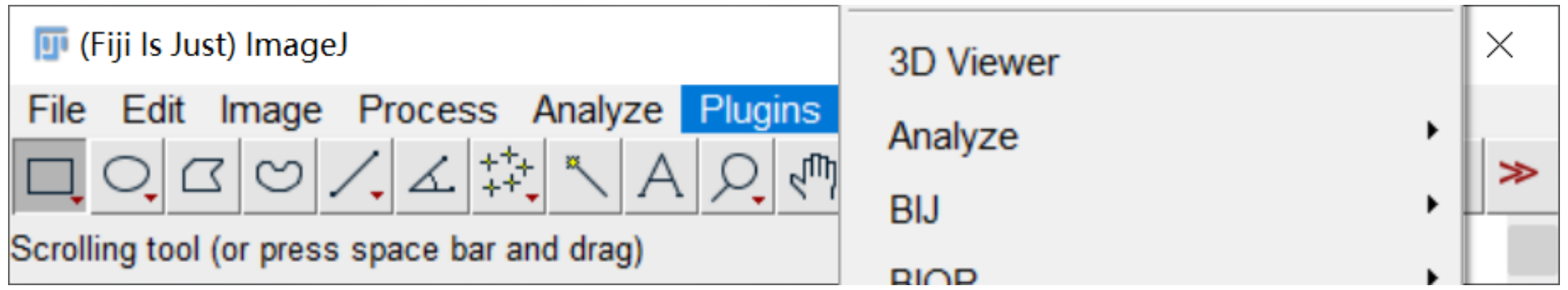

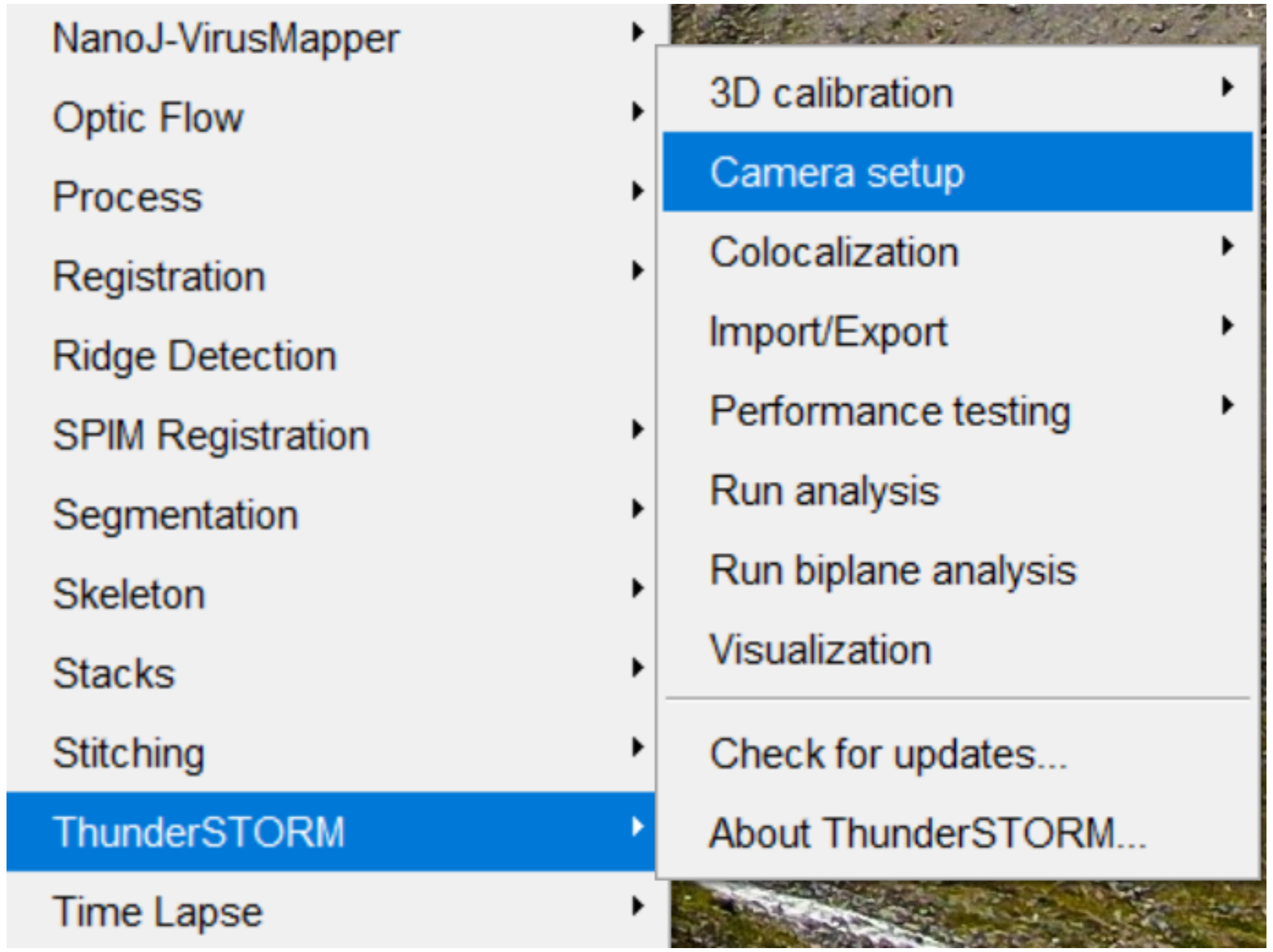

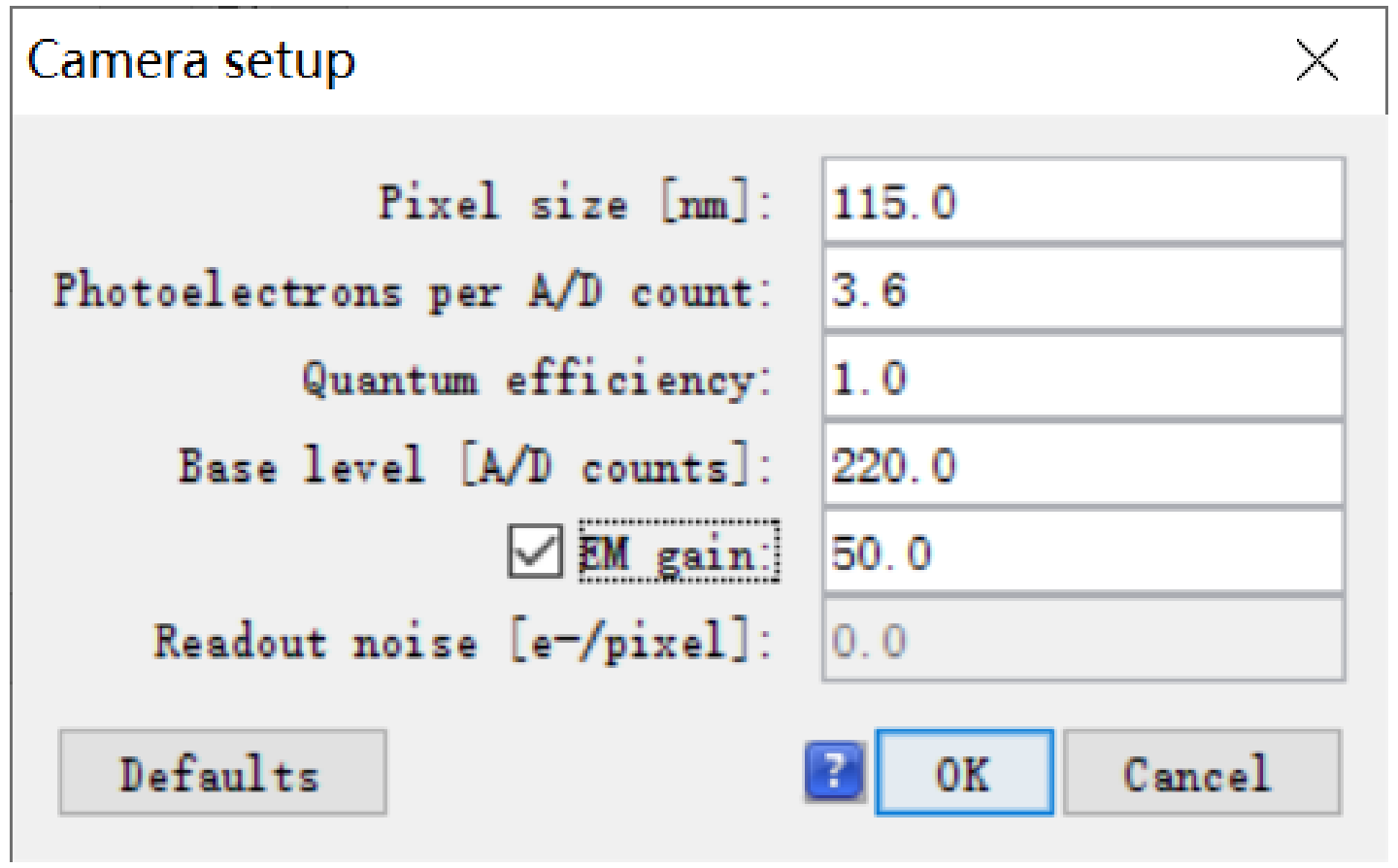

(3) Run the localization analysis

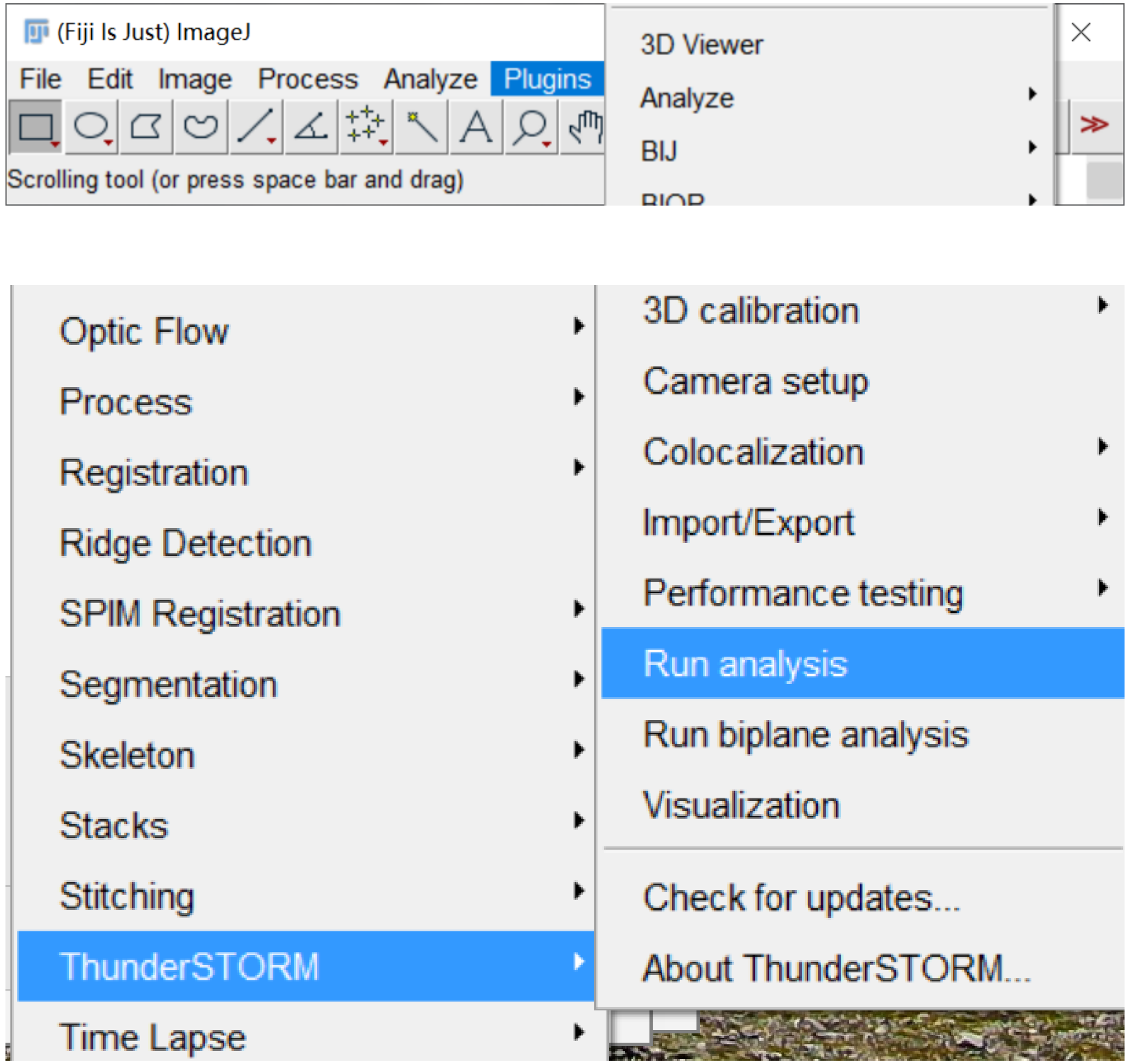

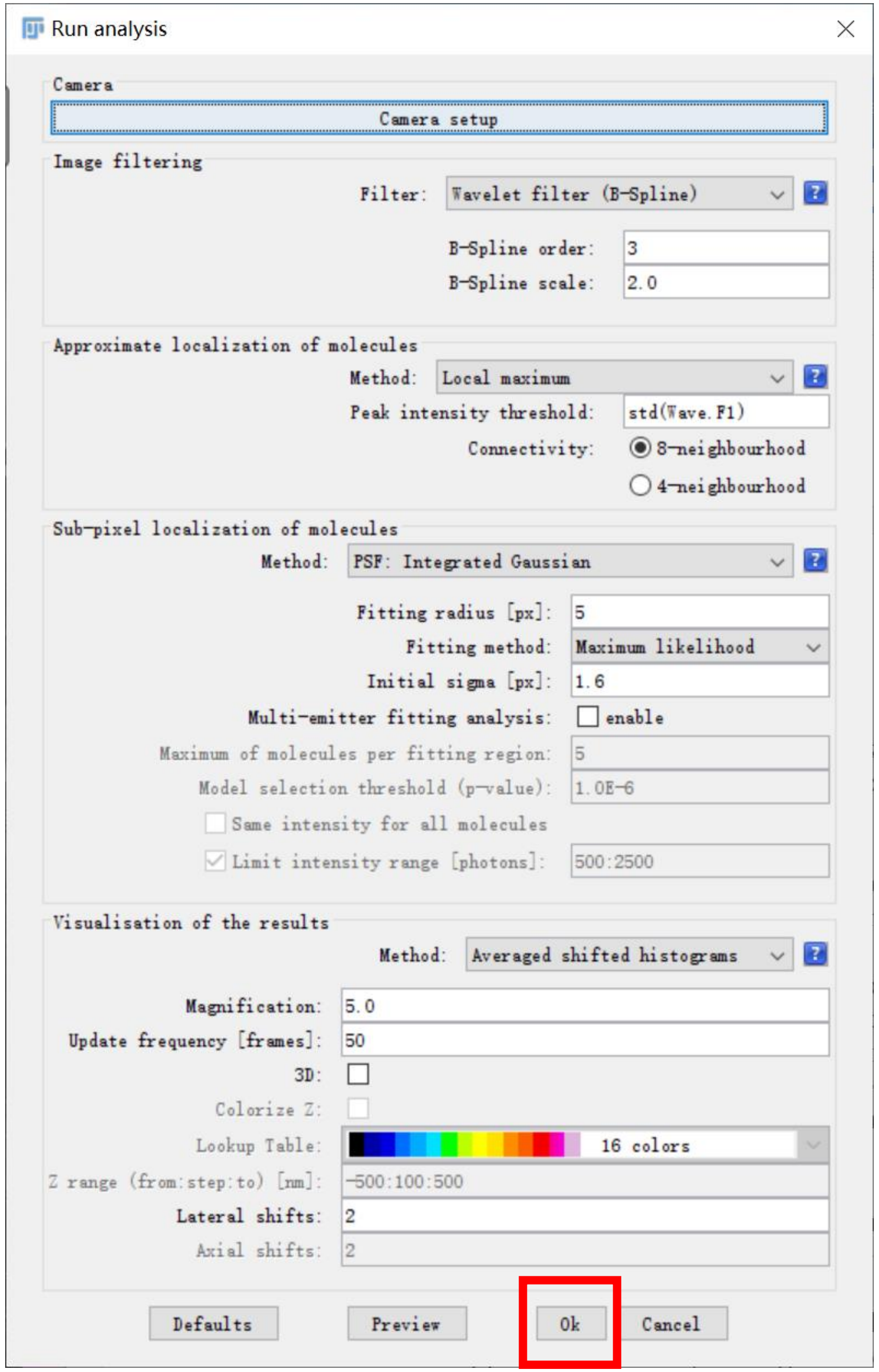

(4) View the localization results

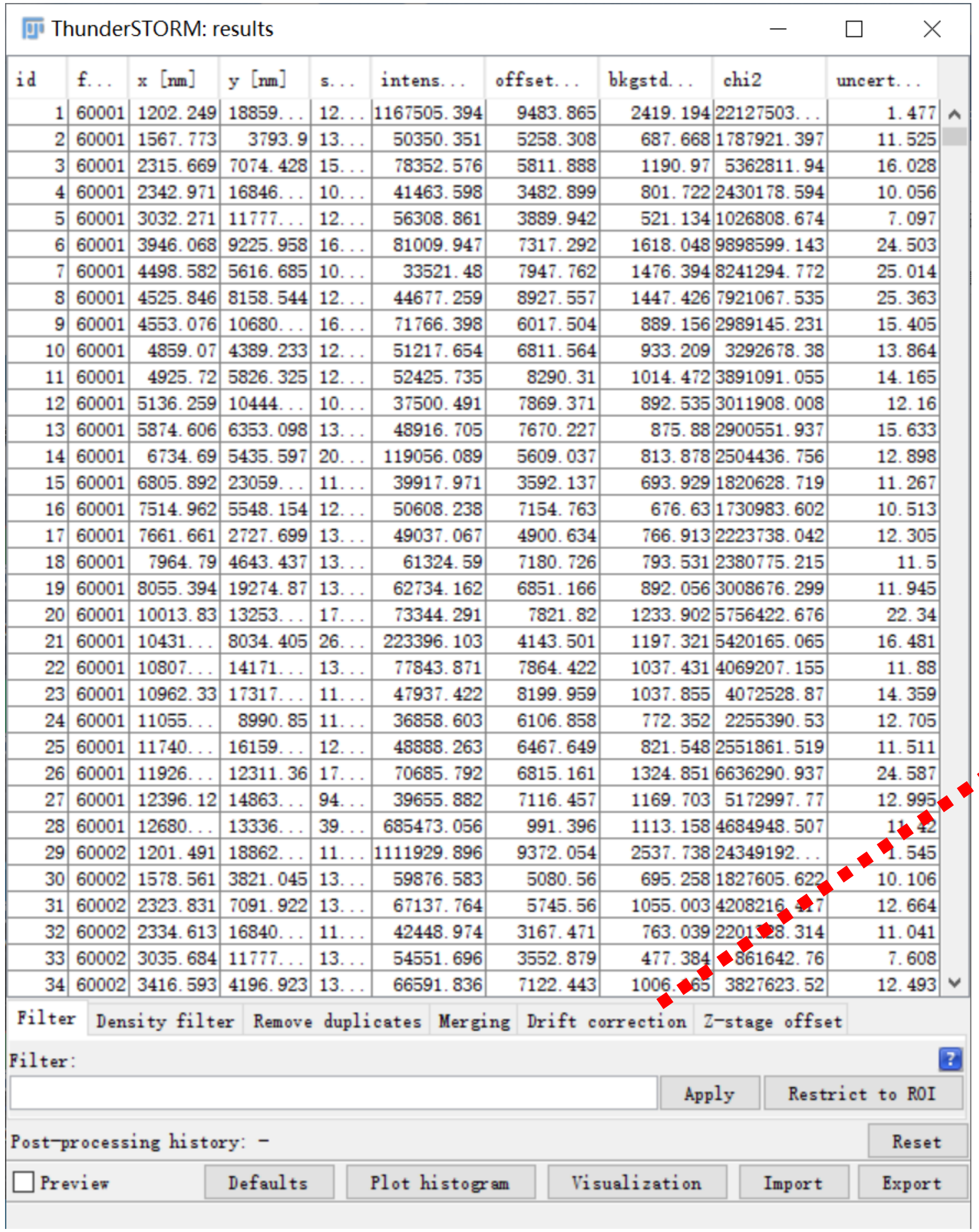

(5) Drift correction:

Fiducial markers --- beads

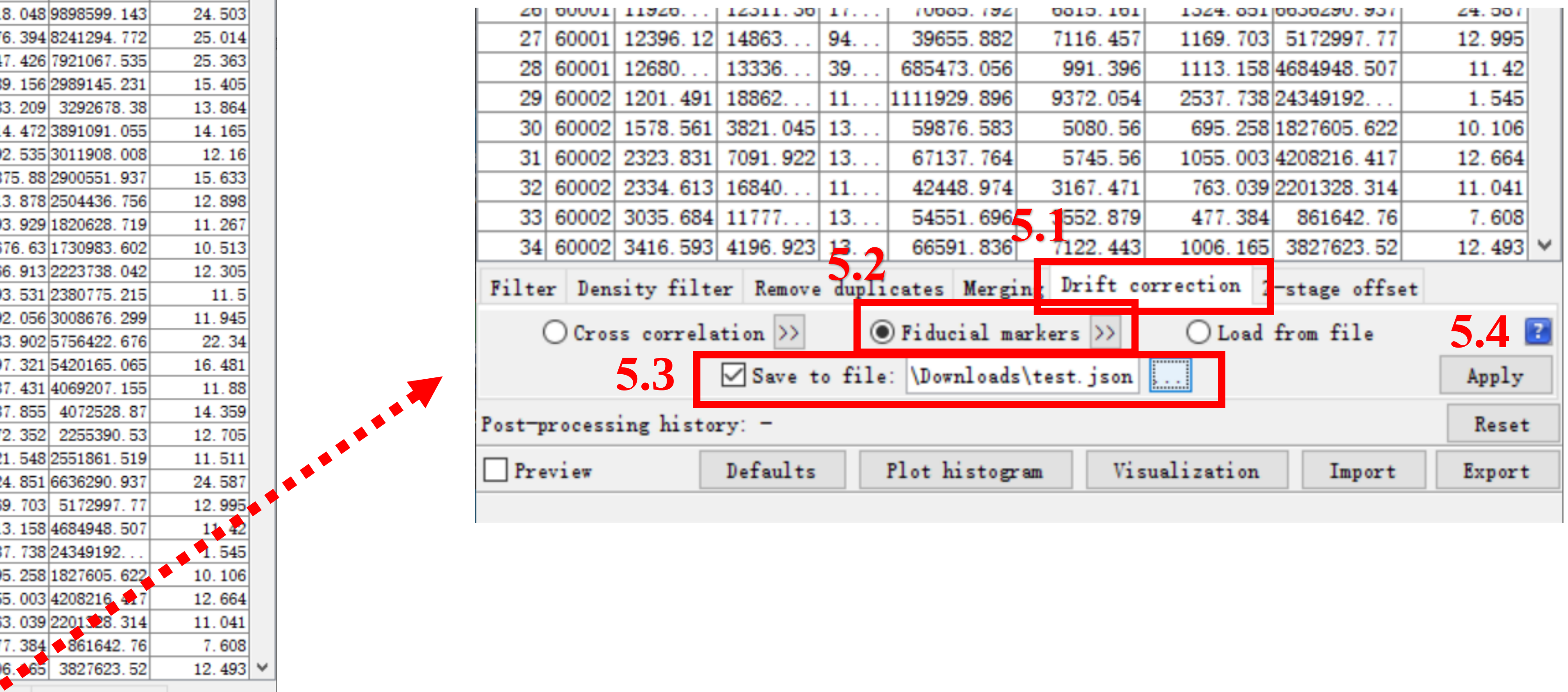

(6) Drift correction:

Load from file --- cells

The localization method is the same as before and will not be repeated here.

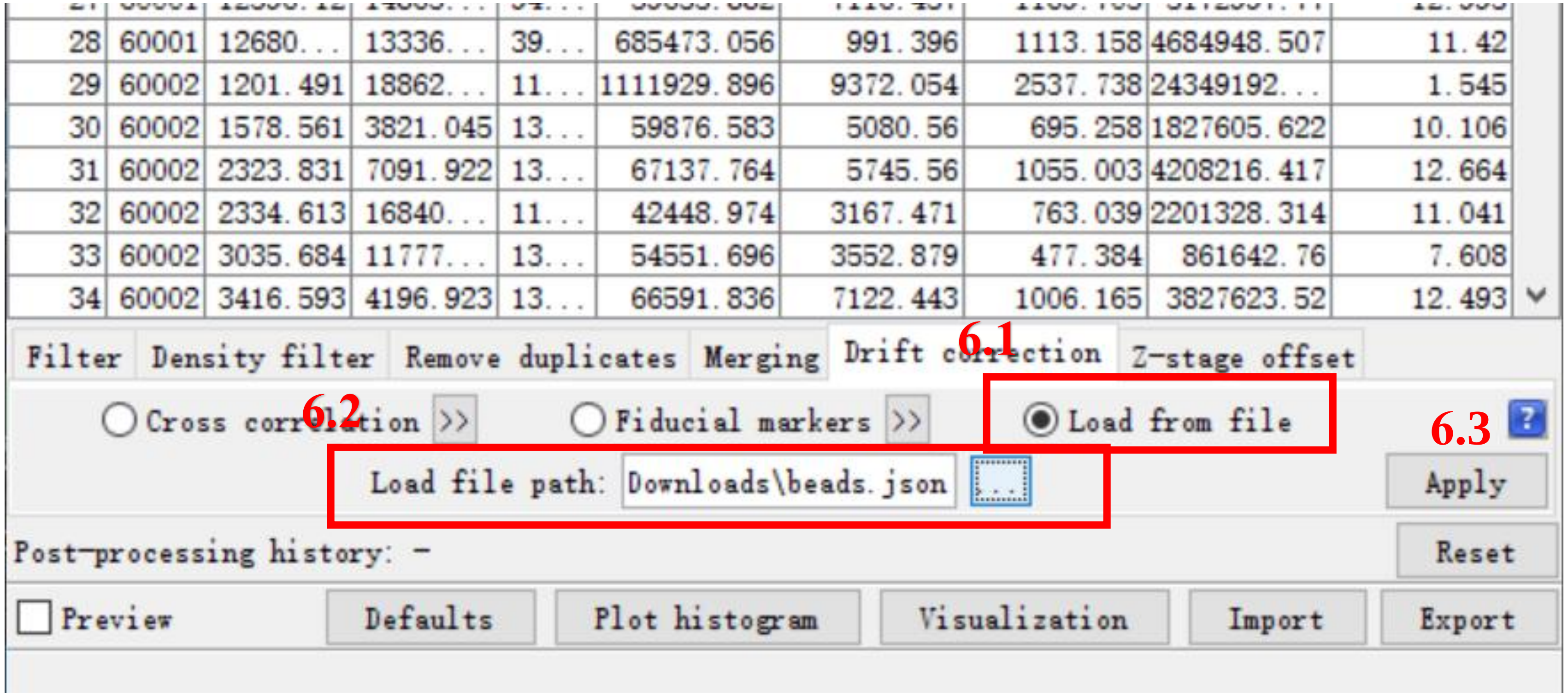

(7) Filter and split channels:

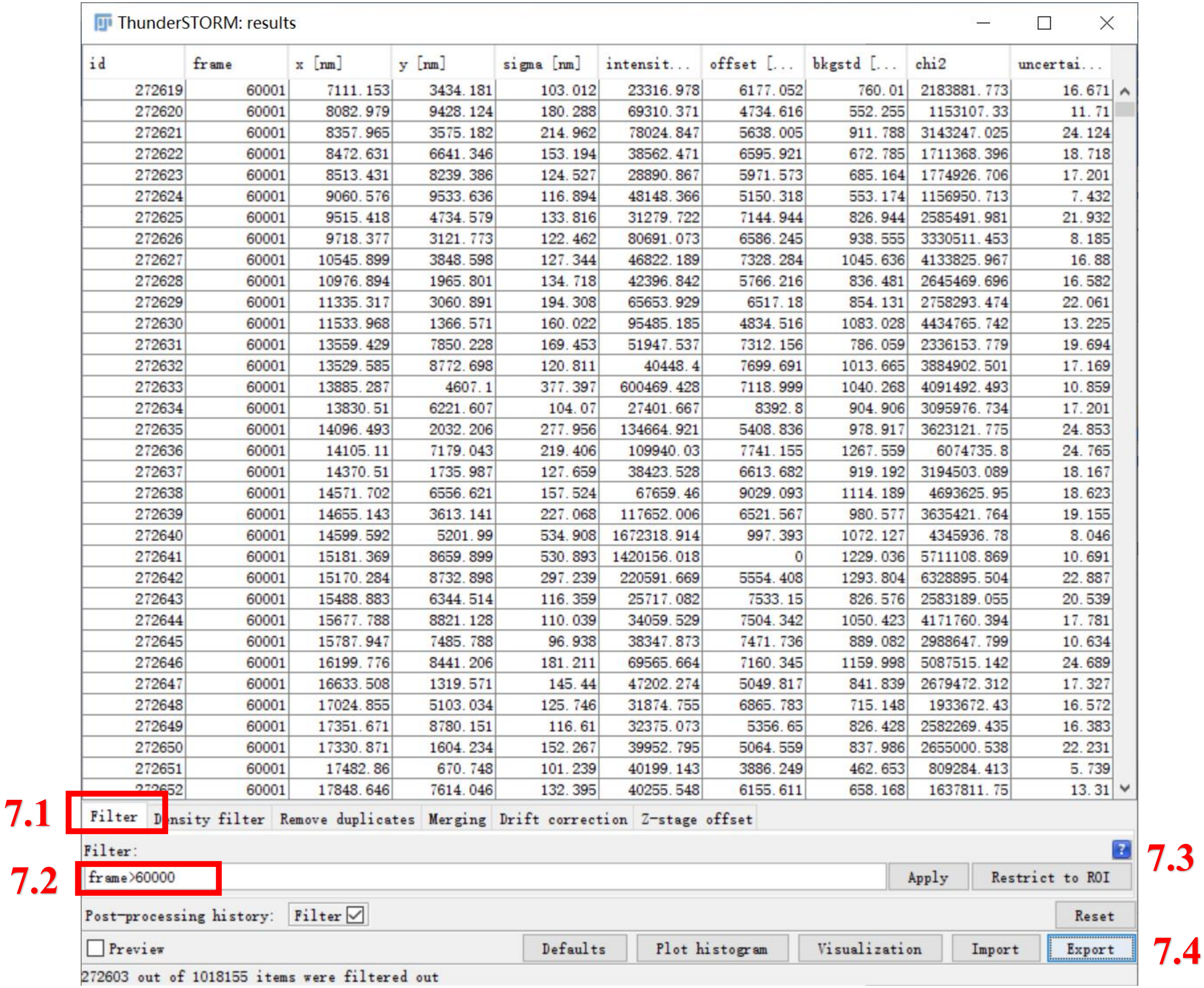

id	frame	x [nm]	y [nm]	sigma [nm]	intensit...	offset [...	bkgstd [...	chi2	uncertai...
272619	60001	7111.153	3434.181	103.012	23316.978	6177.052	760.01	2183881.773	16.671
272620	60001	8082.979	9428.124	180.288	69310.371	4734.616	552.255	1153107.33	11.71
272621	60001	8357.965	3575.182	214.962	78024.847	5638.005	911.788	3143247.025	24.124
272622	60001	8472.631	6641.346	153.194	38562.471	6595.921	672.785	1711368.396	18.718
272623	60001	8513.431	8239.386	124.527	28890.867	5971.573	685.164	1774926.706	17.201
272624	60001	9060.576	9533.636	116.894	48148.366	5150.318	553.174	1156950.713	7.432
272625	60001	9515.418	4734.579	133.816	31279.722	7144.944	826.944	2585491.981	21.932
272626	60001	9718.377	3121.773	122.462	80691.073	6586.245	938.555	3330511.453	8.185
272627	60001	10545.899	3848.598	127.344	46822.189	7328.284	1045.636	4133825.967	16.88
272628	60001	10976.894	1965.801	134.718	42396.842	5766.216	836.481	2645469.696	16.582
272629	60001	11335.317	3060.891	194.308	65653.929	6517.18	854.131	2758293.474	22.061
272630	60001	11533.968	1366.571	160.022	95485.185	4834.516	1083.028	4434765.742	13.225
272631	60001	13559.429	7850.228	169.453	51947.537	7312.156	786.059	2336153.779	19.694
272632	60001	13529.585	8772.698	120.811	40448.4	7699.691	1013.665	3884902.501	17.169
272633	60001	13885.287	4607.1	377.397	600469.428	7118.999	1040.268	4091492.493	10.859
272634	60001	13830.51	6221.607	104.07	27401.667	8392.8	904.906	3095976.734	17.201
272635	60001	14096.493	2032.206	277.956	134664.921	5408.836	978.917	3623121.775	24.853
272636	60001	14105.11	7179.043	219.406	109940.03	7741.155	1267.559	6074735.8	24.765
272637	60001	14370.51	1735.987	127.659	38423.528	6613.682	919.192	3194503.089	18.167
272638	60001	14571.702	6556.621	157.524	67659.46	9029.093	1114.189	4693625.95	18.623
272639	60001	14655.143	3613.141	227.068	117652.006	6521.567	980.577	3635421.764	19.155
272640	60001	14599.592	5201.99	534.908	1672318.914	997.393	1072.127	4345936.78	8.046
272641	60001	15181.369	8659.899	530.893	1420156.018	0	1229.036	5711108.869	10.691
272642	60001	15170.284	8732.898	297.239	220591.669	5554.408	1293.804	6328895.504	22.887
272643	60001	15488.883	6344.514	116.359	25717.082	7533.15	826.576	2583189.055	20.539
272644	60001	15677.788	8821.128	110.039	34059.529	7504.342	1050.423	4171760.394	17.781
272645	60001	15787.947	7485.788	96.938	38347.873	7471.736	889.082	2988647.799	10.634
272646	60001	16199.776	8441.206	181.211	69565.664	7160.345	1159.998	5087515.142	24.689
272647	60001	16633.508	1319.571	145.44	47202.274	5049.817	841.839	2679472.312	17.327
272648	60001	17024.855	5103.034	125.746	31874.755	6865.783	715.148	1933672.43	16.572
272649	60001	17351.671	8780.151	116.61	32375.073	5356.65	826.428	2582269.435	16.383
272650	60001	17330.871	1604.234	152.267	39952.795	5064.559	837.986	2655000.538	22.231
272651	60001	17482.86	670.748	101.239	40199.143	3886.249	462.653	809284.413	5.739
272652	60001	17848.646	7614.046	132.395	40255.548	6155.611	658.168	1637811.75	13.31

(8) Import localization data and visualization

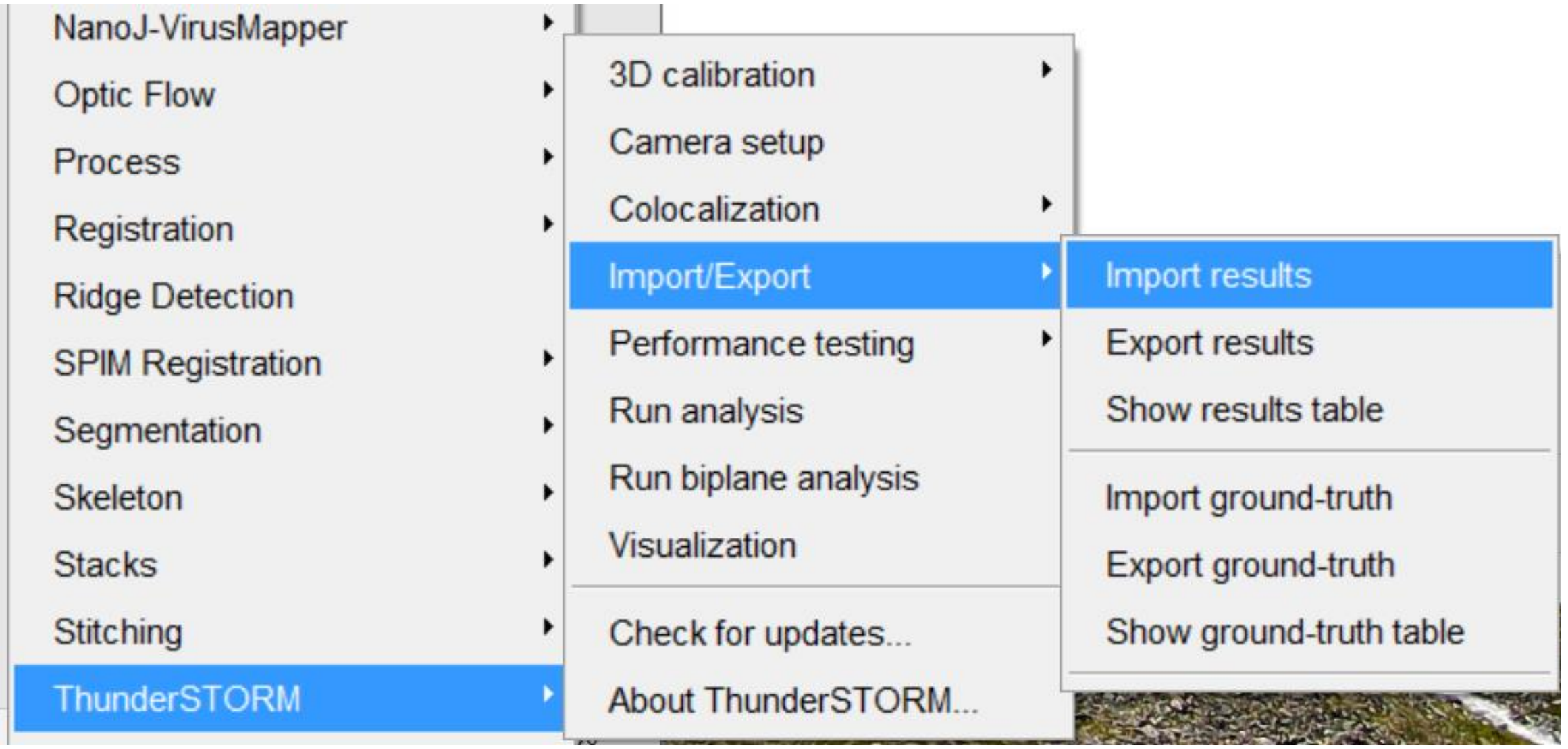

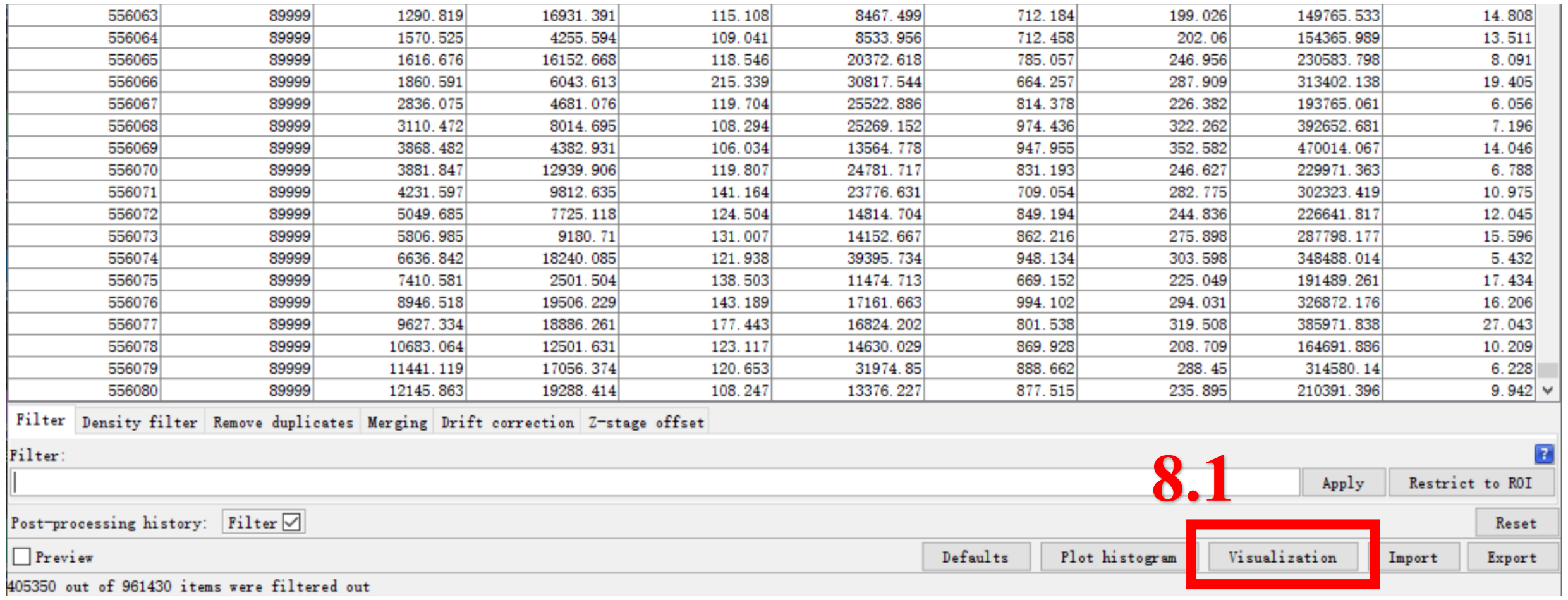

556063	89999	1290.819	16931.391	115.108	8467.499	712.184	199.026	149765.533	14.808
556064	89999	1570.525	4255.594	109.041	8533.956	712.458	202.06	154365.989	13.511
556065	89999	1616.676	16152.668	118.546	20372.618	785.057	246.956	230583.798	8.091
556066	89999	1860.591	6043.613	215.339	30817.544	664.257	287.909	313402.138	19.405
556067	89999	2836.075	4681.076	119.704	25522.886	814.378	226.382	193765.061	6.056
556068	89999	3110.472	8014.695	108.294	25269.152	974.436	322.262	392652.681	7.196
556069	89999	3868.482	4382.931	106.034	13564.778	947.955	352.582	470014.067	14.046
556070	89999	3881.847	12939.906	119.807	24781.717	831.193	246.627	229971.363	6.788
556071	89999	4231.597	9812.635	141.164	23776.631	709.054	282.775	302323.419	10.975
556072	89999	5049.685	7725.118	124.504	14814.704	849.194	244.836	226641.817	12.045
556073	89999	5806.985	9180.71	131.007	14152.667	862.216	275.898	287798.177	15.596
556074	89999	6636.842	18240.085	121.938	39395.734	948.134	303.598	348488.014	5.432
556075	89999	7410.581	2501.504	138.503	11474.713	669.152	225.049	191489.261	17.434
556076	89999	8946.518	19506.229	143.189	17161.663	994.102	294.031	326872.176	16.206
556077	89999	9627.334	18886.261	177.443	16824.202	801.538	319.508	385971.838	27.043
556078	89999	10683.064	12501.631	123.117	14630.029	869.928	208.709	164691.886	10.209
556079	89999	11441.119	17056.374	120.653	31974.85	888.662	288.45	314580.14	6.228
556080	89999	12145.863	19288.414	108.247	13376.227	877.515	235.895	210391.396	9.942

8.2 Adjust parameters

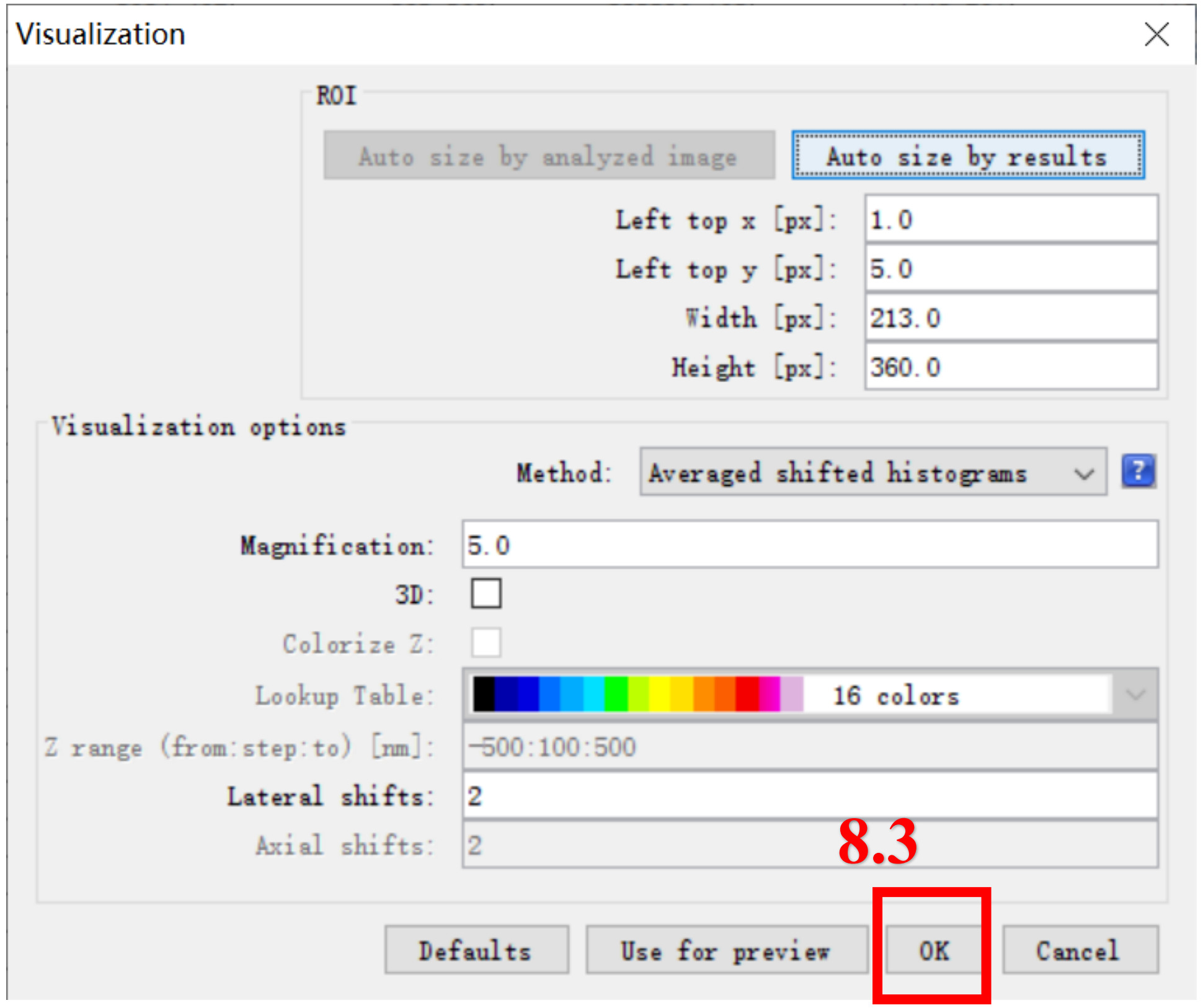

SR-Tesseler operation manual

SR-Tesseler operation manual

(1) Open SR-Tesseler and import the data

1.1

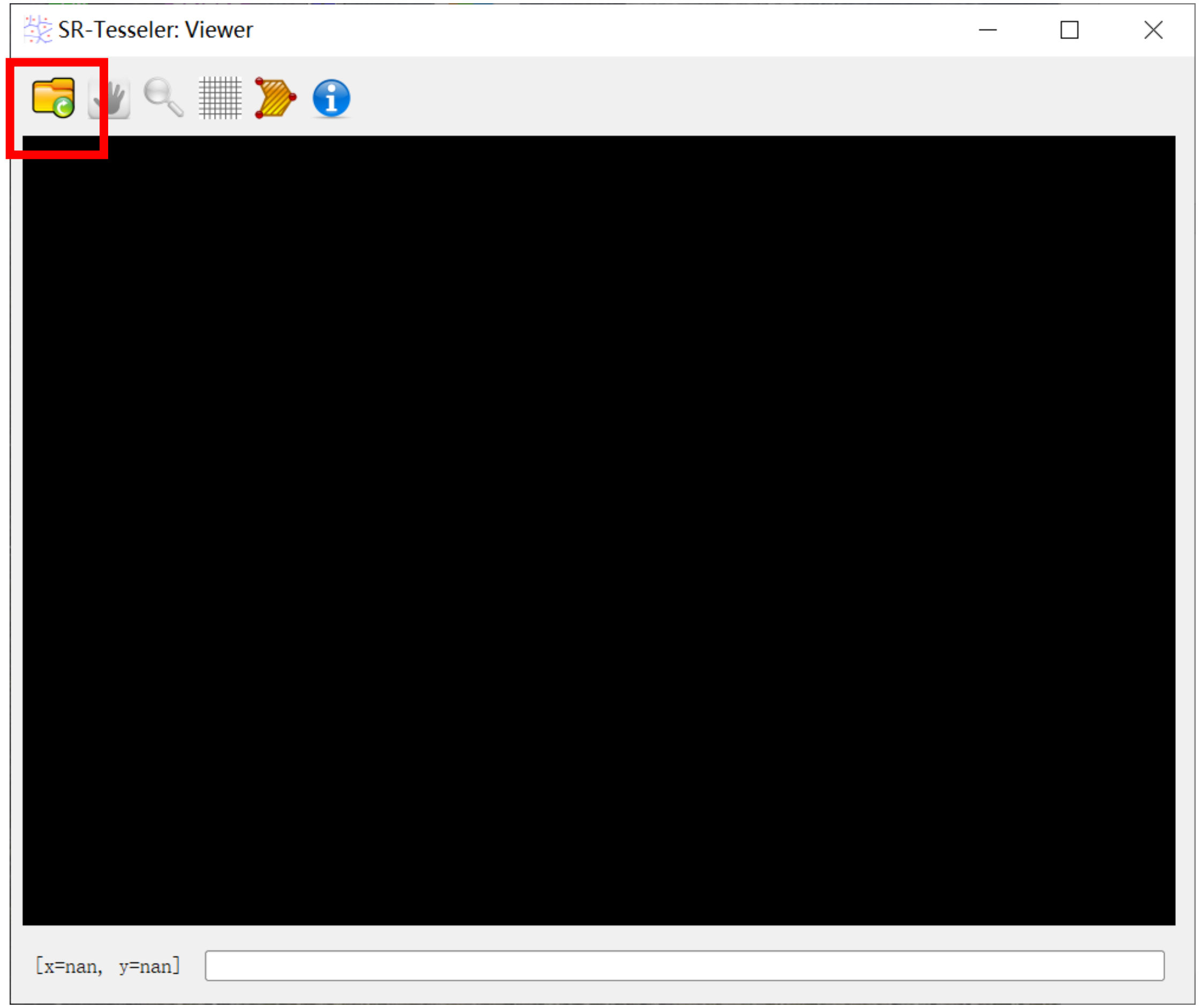

1.2

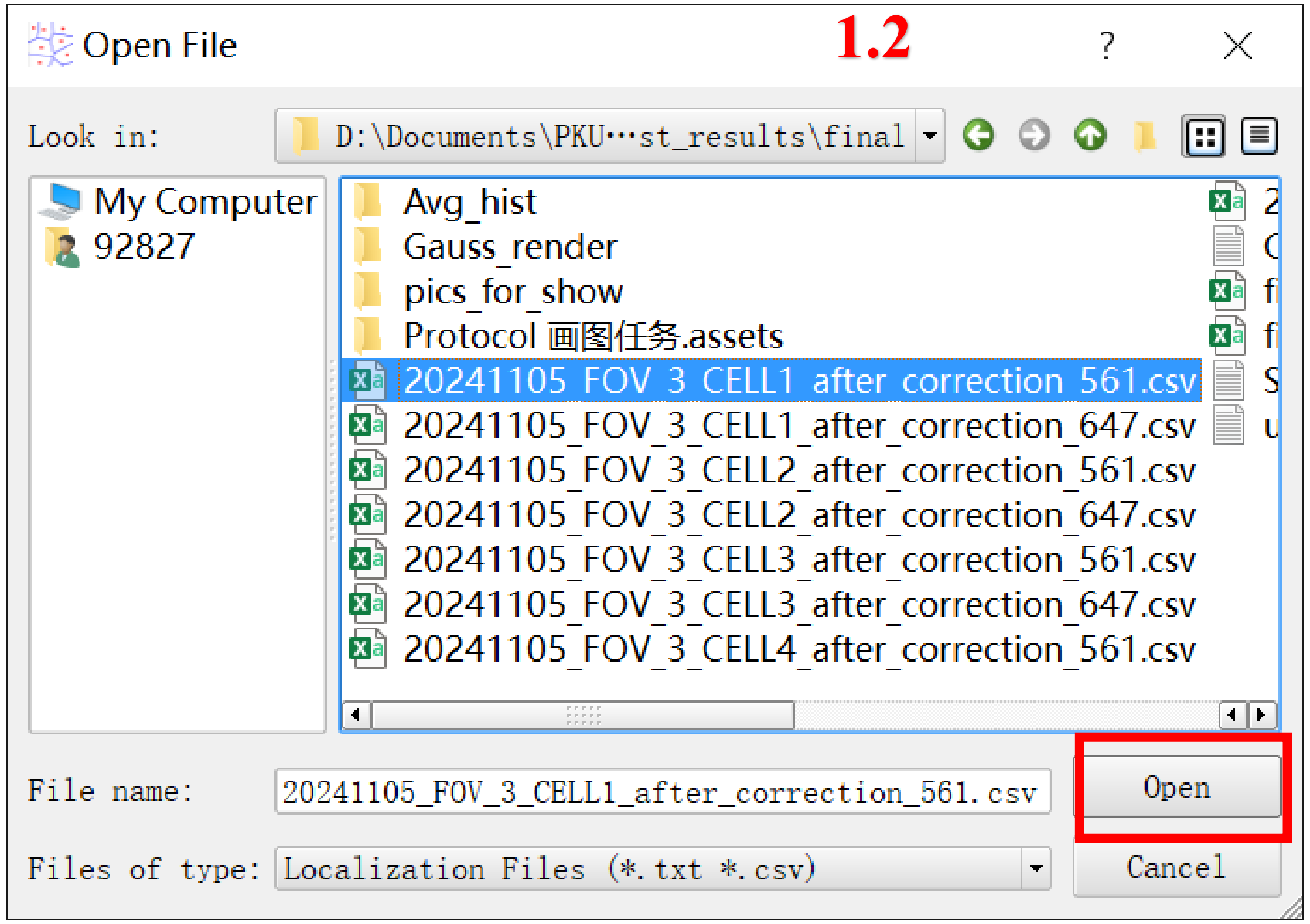

1.3

(2) Adjust the display: select “Log scale” and zoom out in the “SR-Tesseler: Viewer”.

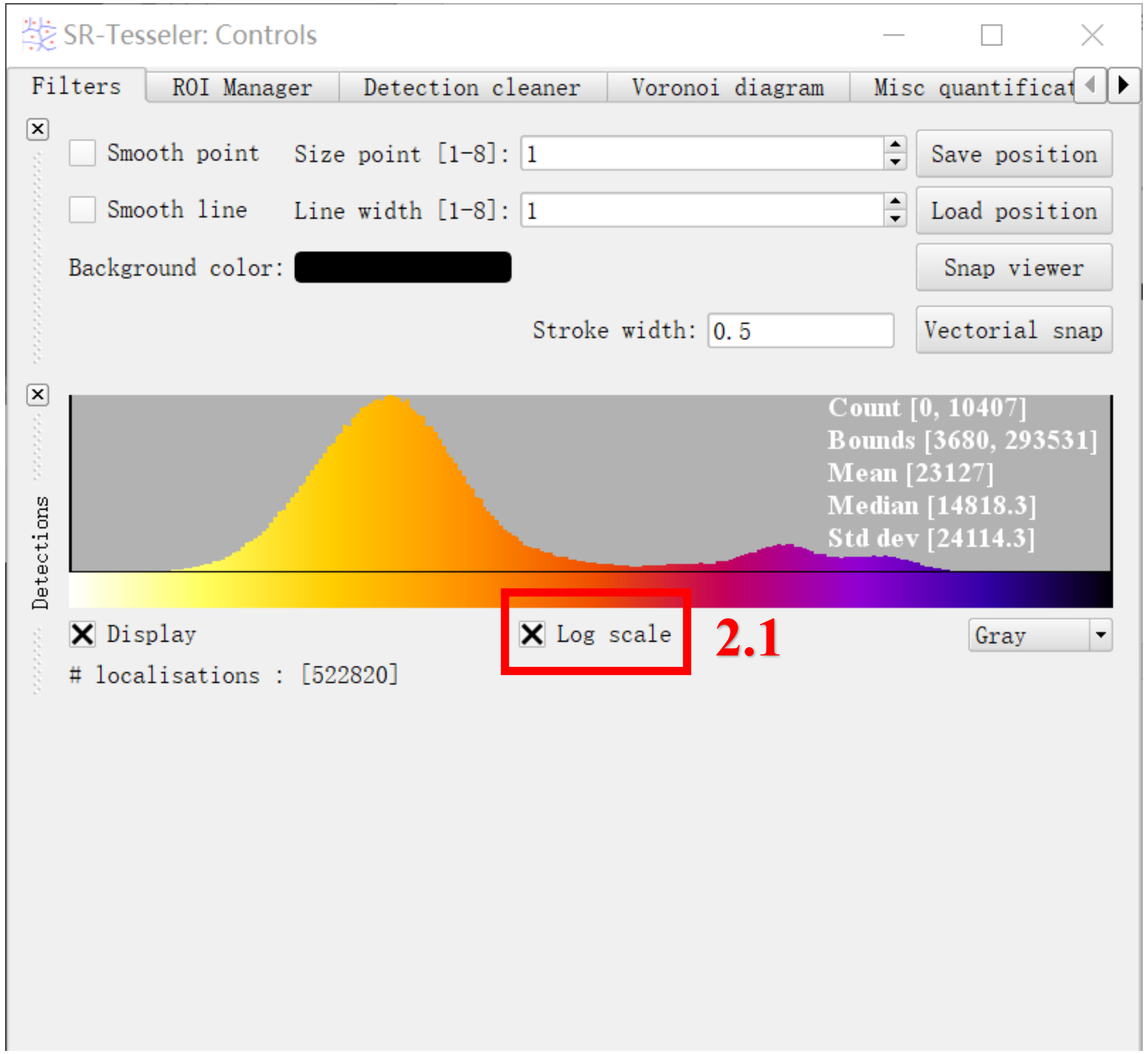

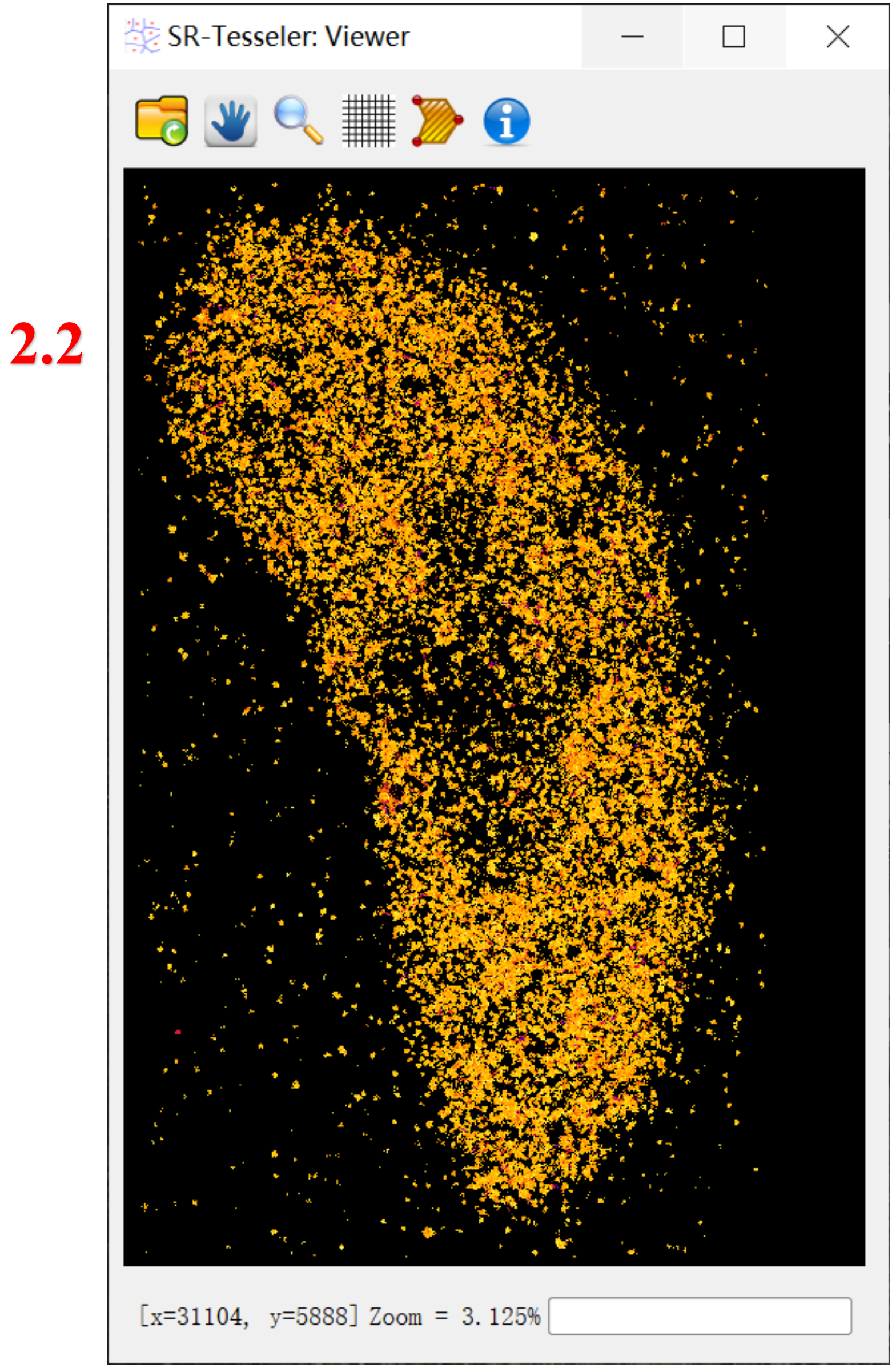

(3) In the "SR-Tesseler: Controls" panel, switch to "Voronoi diagram". Under "Voronoi construction", click "Create polygons" and select "Log scale "below.

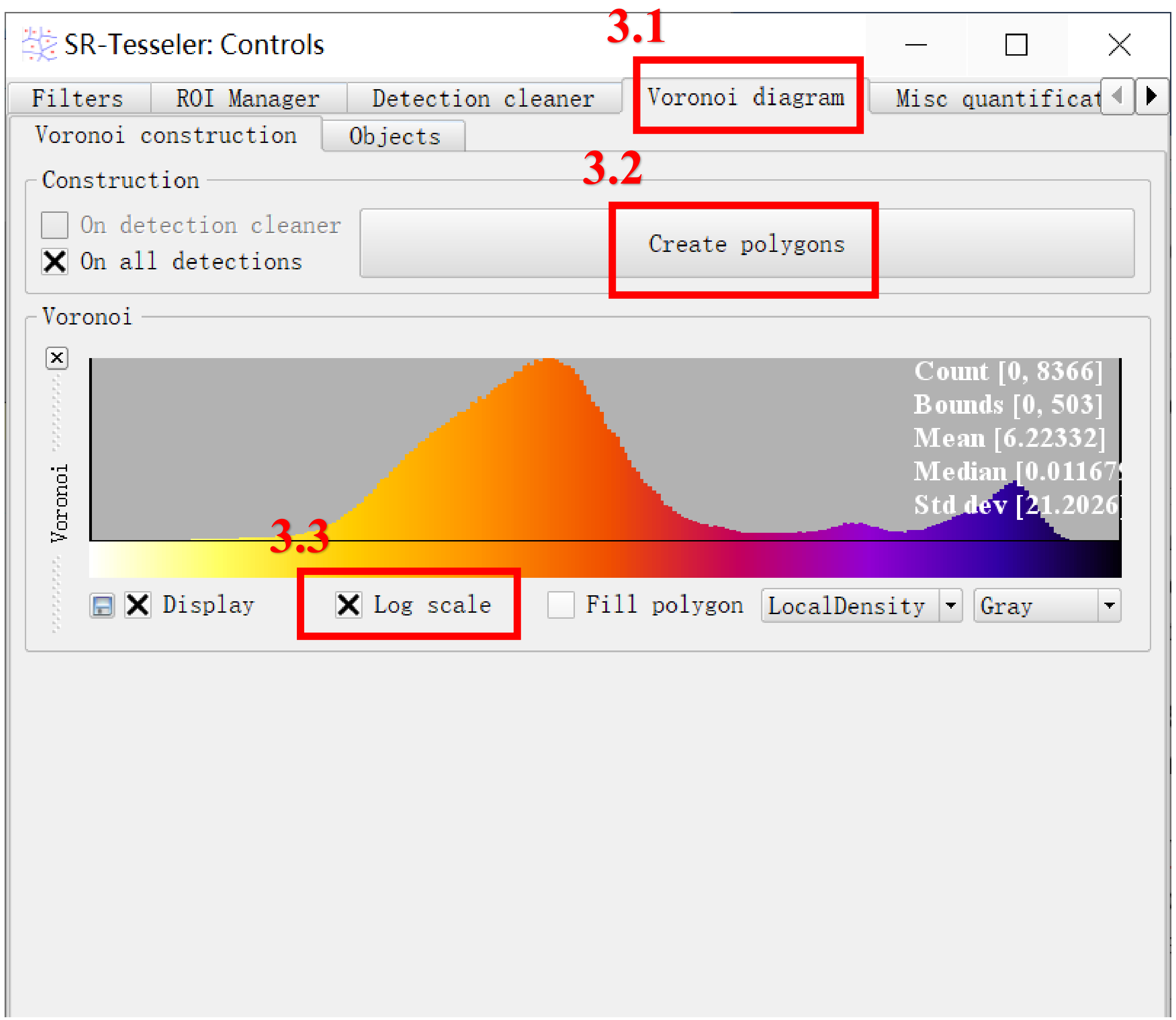

(4) Switch to the Objects tab, configure the relevant parameters, and click Set density factor followed by Create objects to perform object segmentation on the localization results.

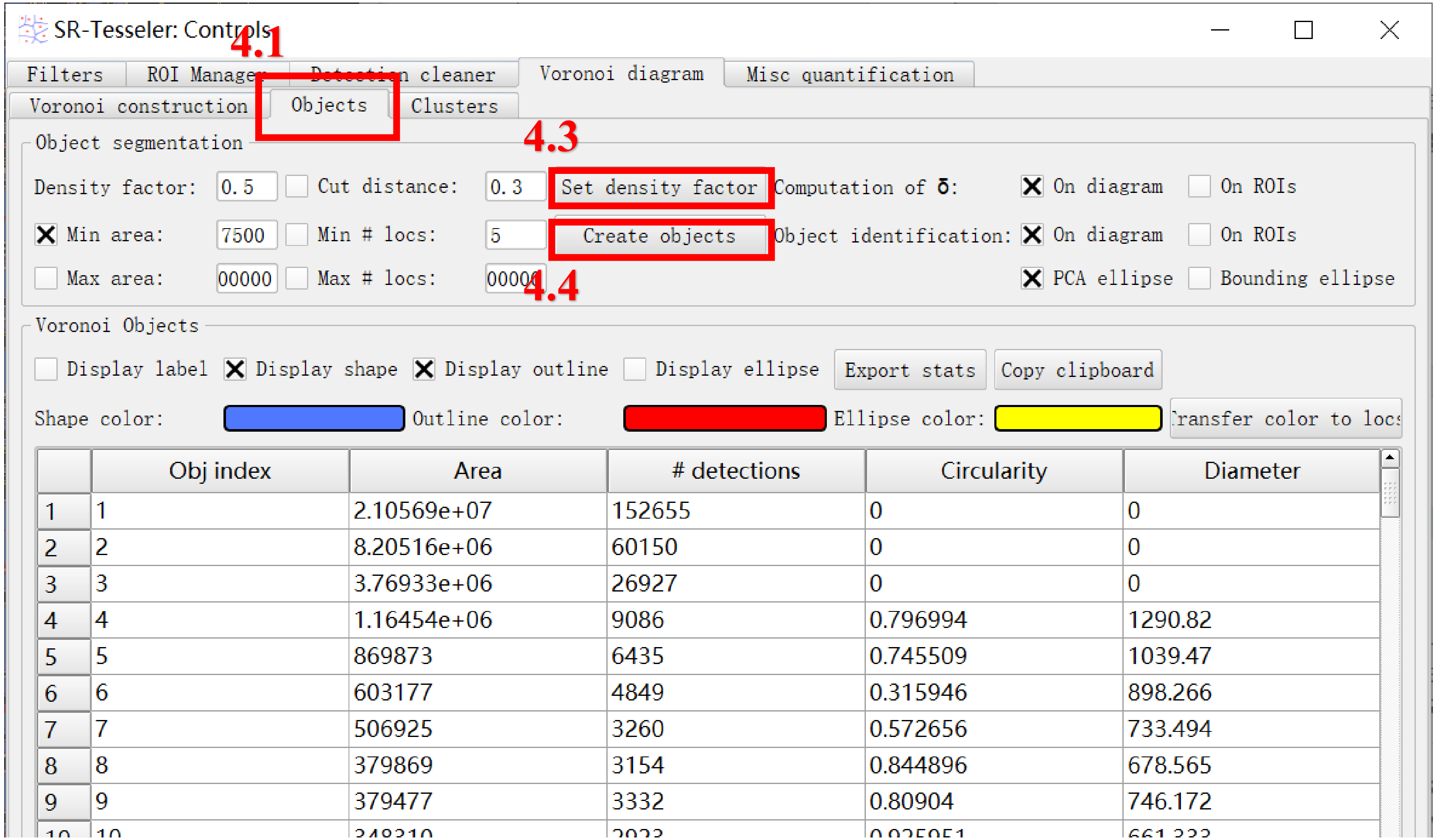

4.2 Adjust parameters

(5) Select Export stats to export the results for subsequent statistical analysis.

To make it look clearer, clear Display label and Display ellipse below.

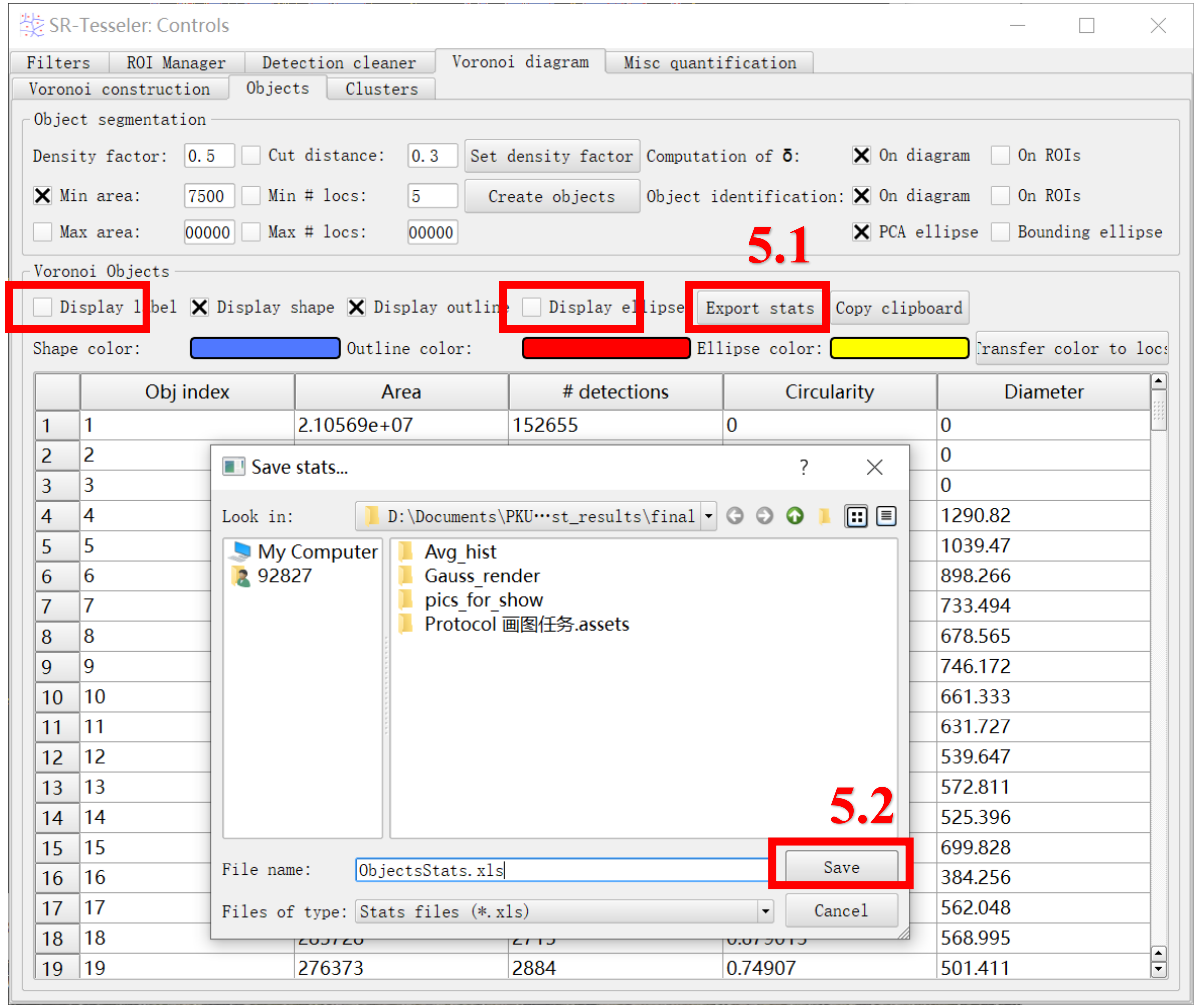

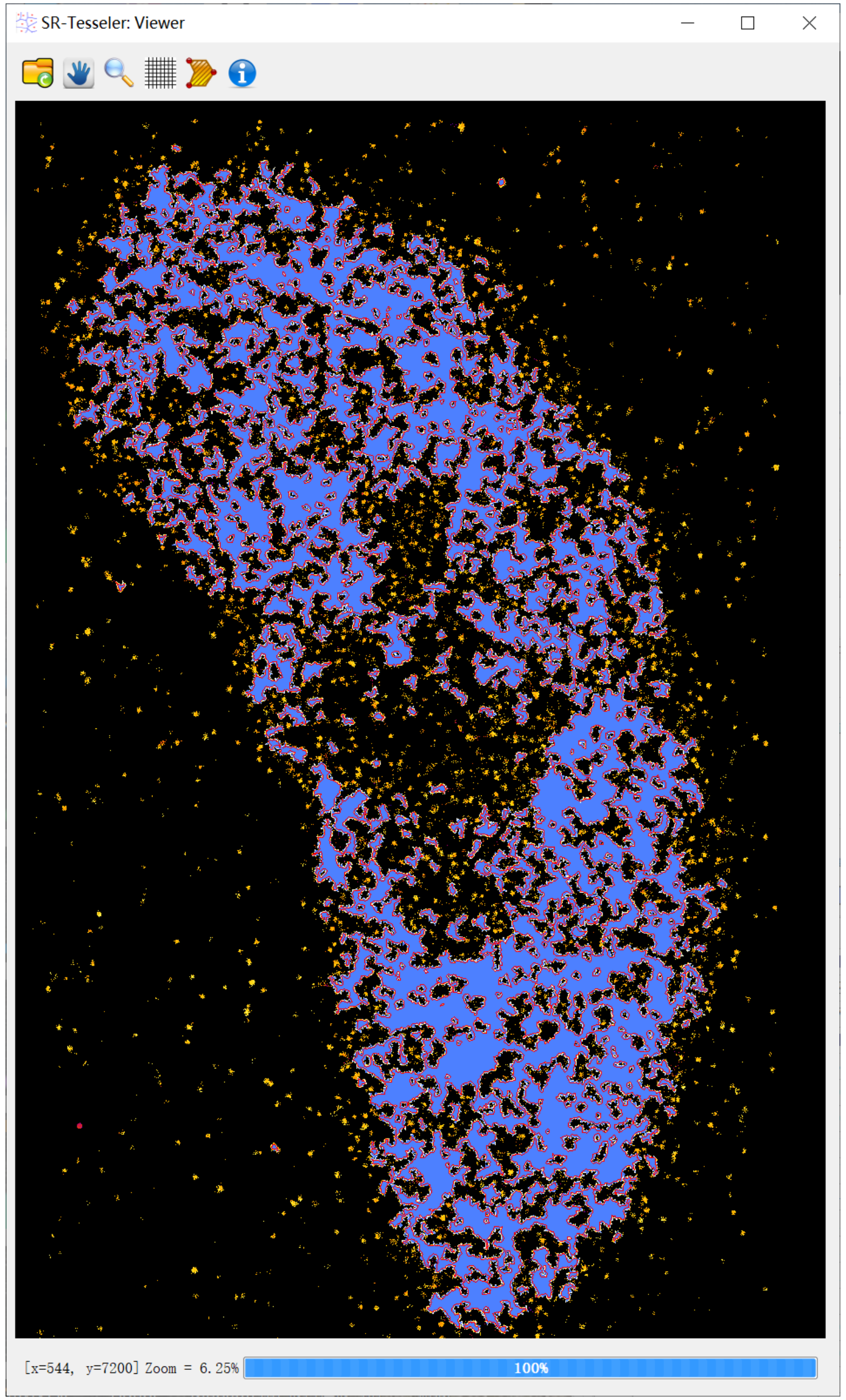

Supplement: Supplementary file 2 [file mmc2.pdf]
